# Tanshinones and diethyl blechnics with anti-inflammatory and anti-cancer activities from *Salvia miltiorrhiza* Bunge (Danshen)

**DOI:** 10.1038/srep33720

**Published:** 2016-09-26

**Authors:** Hongwei Gao, Wen Sun, Jianping Zhao, Xiaxia Wu, Jin-Jian Lu, Xiuping Chen, Qiong-ming Xu, Ikhlas A. Khan, Shilin Yang

**Affiliations:** 1 State Key Laboratory of Quality Research in Chinese Medicine, Institute of Chinese Medical Sciences, University of Macau, Macao, China; 2 National Center for Natural Products Research, and Department of Pharmacognosy, Research Institute of Pharmaceutical Sciences, School of Pharmacy, University of Mississippi, Mississippi 38677, USA; 3 College of Pharmaceutical Science, SooChow University, Suzhou 215123, China

## Abstract

Four novel compounds (**1–4**) as well as fourteen reported compounds (**5–18**) were isolated and purified from *Salvia miltiorrhiza* Bunge (Danshen). The structures of novel compounds were determined by 1D and 2D NMR, HRESIMS data, *etc.* The anti-inflammatory properties of all the compounds on RAW264.7 macrophages and their cytotoxicity on H1299 and Bel-7402 cell lines coupled with a structure-activity relationship (SAR) were investigated. Compound **4** demonstrated the best anti-inflammatory activity and was chosen for further research. Compound **4** greatly suppressed secretion of nitric oxide (NO), tumor necrosis factor (TNF)-α and interleukin-6 (IL-6) in the RAW264.7 macrophages stimulated by LPS. Additionally, the protein expression of inducible nitric oxide synthase (iNOS) and cyclooxygenase-2 (COX-2) was decreased and the nuclear translocation of NF-κB was attenuated after treatment with compound **4** *in vitro*. Compound **4** was able to dramatically inhibit LPS-induced activation of JNK1/2 and ERK1/2 and remarkably disrupted the TLR4 dimerization in LPS-induced RAW264.7 macrophages. Thus, the new compound **4** suppressed LPS-induced inflammation partially is due to the blocking TLR4 dimerization. In addition, the anti-cancer activity investigation indicated that most of isolated compounds exhibited cytotoxicity and the SAR analysis showed that the intact D ring was indispensable and unsaturated D ring played vital role.

The inflammatory process is a self-protective response against external harmful ingression like pathogens, damaged cells or irritants^1^,^2^. However, aberrant inflammation, leads to the etiology of inflammatory disorders, such as asthma, rheumatoid arthritis (RA), Crohn’s disease (CD) and inflammatory bowel disease (IBD)^3^,^4^. The activation of transcriptiona factor NF-κB has been widely accepted for its center role in the initiation and amplification of the inflammatory response^5^,^6^,^7^. Many genes, particularly those involved in inflammation including inflammometer (NO), cytokines (TNF-α and IL-6), COX-2, etc. are regulated through NF-κB signal pathway^8^,^9^.

*Salvia miltiorrhiza* Bunge (Danshen), well known for its highly medicinal properties in treating of heart and vascular diseases, has also been explored extensively as a source for treatment of chronic renal failure, Alzheimer’s disease or various types of hepatitis^10^,^11^. Furthermore, Danshen is the first traditional Chinese medicine that is documented in the United States Pharmacopeia^12^. As it stands now, quite a few natural products isolated from Danshen, such as tanshinone IIA, salvianolic acids, *etc.* gain the approval from the state food and drug administration of China (SFDA) for the use as therapeutic agents in caridovascular diseases. Furthermore, the Danshen capsule, Danshen pill, FuFangDanshen drop pill, tanshinone capsules, which contain one or more herbs including Danshen, are widely prescribed in clinical in China. Of note, FuFangDanshen drop pill has been entered into clinical trial in United States.

Danshen has been one of the most widely investigated herbs worldwide at present. To date, over 20,000 publications related to Danshen could be retrieved from the SciFinder database. Furthermore, more than 100 compounds have been identified from Danshen. Classified in terms of structural characteristics and chemical properties, the compounds isolated from Danshen can be categorized as water-soluble and lipid-soluble constituents^13^. Water-soluble constituents, including salvianolic A-G or lithospermic acid B, mainly exhibit cardiovascular protective activities^14^,^15^. While the lipid-soluble constituents, including tanshinone IIA, dihydrotanshinone I, or cryptotanshinone, show remarkable properties of anti-cancer and anti-inflammation^16^,^17^,^18^,^19^. In this study, accompanied by fourteen known compounds (**5–18**), four novel compounds (**1–4**) (Fig. 1) were obtained from Danshen. Four compounds were structurally elucidated and the isolated compounds were used to investigate their anti-inflammatory and anticancer activities.

## Results and Discussions

**Figure 2:**
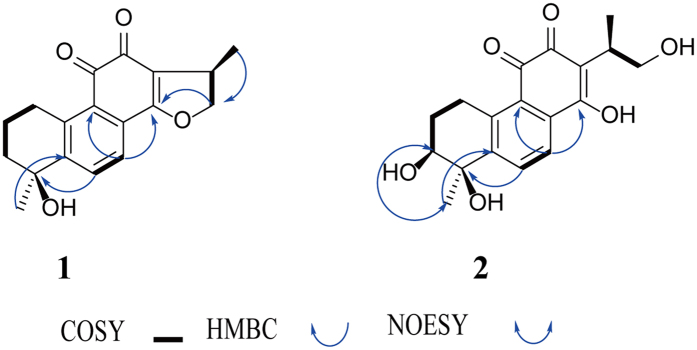
Key COSY, NOESY and HMBC correlations of compounds 1 and 2.

Compound **1** was red powder with molecular formula as C_18_H_18_O_4_ determined by HRESIMS at *m/z* 299.1272 [M + H]^+^. The IR adsorptions 1637, 2967 and 3368 cm^−1^ indicated the presence of methyl, hydroxyl and carbonyl groups, respectively. ^1^H NMR data revealed the presence of one tertiary methyl (*δ*_H_ 1.69, 3H, s), one secondary methyl (*δ*_H_ 1.29, 3H, d, *J* = 6.8 Hz), and AB pattern for ortho-aromatic protons (*δ*_H_ 7.65, 1H, d, *J* = 7.5; *δ*_H_ 8.32, 1H, d, *J* = 7.5) (Table 1). All 18 carbons of **1** were resolved as individual carbon resonances (Table 1) by means of ^13^C NMR spectrum and classified by DEPT and HSQC spectrums showing as the presence of two methyls, four sp^3^ methylenes (one oxygenated), one quaternary carbon (oxygenated), four double bonds, and two carbonyls (*δ*_C_ 184.9, 176.6). In terms of the NMR data of cryptotanshinone and tanshinol B^20^,^21^, compound **1** had a similar structure with them. As indicated by spectrum results from ^1^H-^1^H COSY experiment (Fig. 2), H-2 showed correlations with H-1 and H-3, respectively, while H-15 had correlations with H-16 and H-17, respectively, and H-6 had a correlation with H-7. The hydroxyl group was located at C-4, which was supported by the key HMBC correlations of C-4 and H-6/H-2. The stereochemistry of compound **1** was elucidated by NOESY spectrum (Fig. 2) and literatures published^20^,^21^. In comparison of NMR data of cryptotanshinone and tanshinol B, H-15 was α-oriented and 15- methyl group was *β*-oriented, 4-methyl group was α-oriented and 4-hydroxyl group was *β*-oriented. Therefore, the structure of compound **1** was identified as (1R,6S)-6-hydroxy-1,6-dimethyl-1,2,6,7,8,9hexahydrophenanthro[1,2-b]furan-10,11-dione.

Compound **2**, isolated as orange powder, was elucidated as C_18_H_20_O_6_ in accordance with the HRESIMS at *m/z* 333.1329 [M + H]^+^. The IR adsorptions 1638, 2974 and 3379 cm^−1^ indicated the presence of carbonyl, methyl and hydroxyl groups, respectively. Its ^1^H NMR and ^13^C NMR data (Table 1) showed high similarity with compound **1** bar major change of the chemical shifts of C-3 and C-14. *δ*_C_ 39.2 (C-3) in compound **1** was substituted by *δ*_C_ 73.0 (C-3) in compound **2**. The change of chemical shift of C-3 in compound was induced by its proton (H-3) replaced by hydroxyl. *δ*_C_ 169.8 (C-14) in compound **1** was in lieu of *δ*_C_ 173.3 (C-14) in compound **2**. The change of chemical shift of C-14 in compound **2** was induced by the break of D ring. In terms of HMBC spectrum of compound **1**, there are clear correlated signals between C-14 (*δ*_C_ 169.8) and H-16 (*δ*_H_ 4.20, 4.76). However, the correlated signals between C-14 and H-16 in HMBC spectrum of compound **2**. Collectively, these data indicated the D ring was broken. In terms of compound **1**, 4-methyl group was α-oriented. From NOESY spectrum (Fig. 2), there is a correlation between H-22 (*δ*_H_ 1.45) and H-3 (*δ*_H_ 3.82), which indicated that H-3 was α-oriented and OH-3 was *β*-oriented. Therefore, the structure of compound **2** was identified as (7S,8R)-1,7,8-trihydroxy-2-((R)-1-hydroxypropan-2-yl)-8-methyl-5,6,7,8-tetrahydrophenanthrene-3,4-dione.

Compound **3** was obtained as orange red powder. Its chemical formula was calculated as C_19_H_22_O_4_ in concert with the HRESIMS at *m/z* 315.1579 [M + H]^+^. The IR adsorptions 2976 and 3392 cm^−1^ indicated that the presence of methyl and hydroxyl groups, respectively. Its ^1^H NMR and ^13^C NMR data (Table 1) showed high similarity with compound **2** except change of chemical shifts of C-4 (*δ*_C_ 34.8), C-11 (*δ*_C_ 167.2), and C-12 (*δ*_C_ 165.7). The groups of C-4 were replaced by a gem-dimethyl group. The carbonyl groups were reduced to hydroxyls in compound **3**. Comparing with compound **1** and compound **2,** H-3 was α-oriented and OH-3 *β*-oriented and H-15 was α-oriented and CH_3_-15 *β*-oriented. Therefore, the structure of compound **3** was identified as (1R,7S)-1,6,6-trimethyl-1,2,6,7,8,9-hexahydrophenanthro- [1,2-b]furan-7,10,11-triol.

Compound **4** was isolated as orange powder with chemical formula deduced as C_22_H_22_O_8_ from the HRESIMS at *m/z* 415.1381 [M + H]^+^. Its ^1^H NMR and ^13^C NMR data (Table 2) showed similarity with blechnic acid^22^. Comparing with the NMR data of blechnic acid, four more carbon signals C-10 (*δ*_C_ 62.0), C-11 (*δ*_C_ 14.2), C-21 (*δ*_C_ 60.4) and C-22 (*δ*_C_ 14.6) were appeared in the carbon spectrum of compound **4**. From COSY spectrum, there were correlations between H-10 (*δ*_H_ 4.20) and H-11 (*δ*_H_ 1.12), H-21 (*δ*_H_ 4.27) and H-22 (*δ*_H_ 1.21). In addition, according to HMBC, there were correlations between C-9 (*δ*_C_ 172.2) and H-10 (*δ*_H_ 4.20), C-20 (*δ*_C_ 167.3) and H-21 (*δ*_H_ 4.27). Therefore, these data indicated that the two carboxyl groups of blechnic acid had an esterification. From NOESY spectrum, there was a correlation between H-7 (*δ*_H_ 6.46) and H-8 (*δ*_H_ 4.92), which indicated the two protons were *β*-oriented. Therefore, the structure of compound **4** was identified as ethyl (2S,3R)-2-(3,4-dihydroxyphenyl)-4-((E)-3-ethoxy-3-oxoprop-1-en-1-yl)-7-hydroxy-2,3-dihydrobenzofuran-3-carboxylate.

### Isolated Compounds Exhibited The Anti-inflammatory Activities to LPS-Induced RAW264.7 Cells

**Figure 3:**
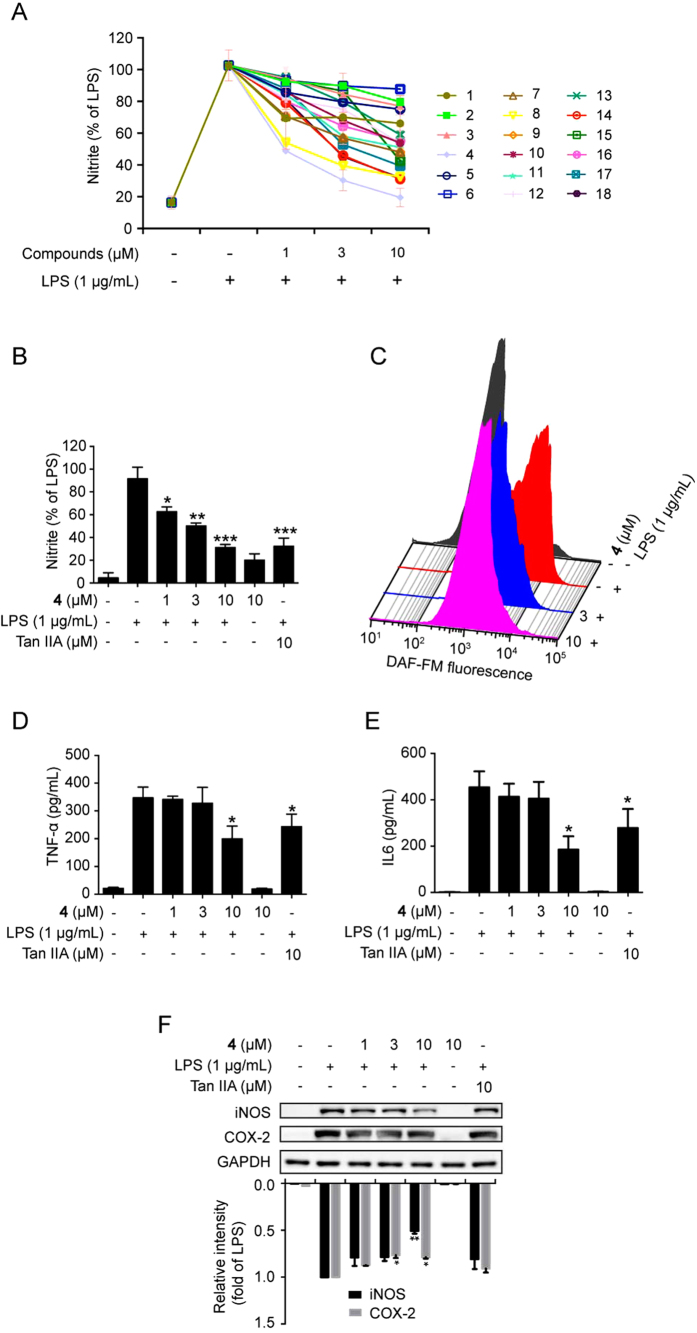
The anti-inflammatory activities of the isolated compounds. (**A**) Cells were pretreated with compounds for 1 h and then stimulated with/without LPS (1 μg/mL) for 24 h. The nitrite production was determined by Griess assay. (**B**) The effect of compound **4** and Tan IIA on nitrite production was determined by Griess assay. *p < 0.05, **p < 0.01, and ***p < 0.001 versus LPS-treated group. (**C**) NO production was measured by flow cytometry with DAF-FM (3 μM). (**D**,**E**) Cells were pretreated with compound **4** for 1 h and then stimulated with/without LPS (1 μg/mL) for 24 h. The ELISA assay was employed to detect levels of TNF-α and IL-6 in the culture medium. *p < 0.05 versus the LPS-treated group. (**F**) Cells were pretreated with compound **4** as aforementioned and the expression of iNOS and COX-2 was determined by Western blotting. Tan IIA, tanshinone IIA.

To date, there are around 60 tanshinones isolated and identified from Danshen^23^. However, only a few of them, such as tanshinone IIA, cryptotanshinone, were thoroughly investigated for their bioactivities. Although the anti-inflammatory activity of tanshinone IIA and cryptotanshinone has been well established^24^,^25^, most of the known compounds isolated by our group such as compounds **5, 6, 7, 9, 10, 11, 12, 13** and **14** have not been reported. Synthesized endogenously from L-arginine by nitric oxide synthases (NOSs), NO becomes an inflammometer to modulate important cellular signaling involved in immunity and inflammation^26^. LPS-induced NO production in RAW264.7 macrophages has been considered as the convenient and credible method for anti-inflammation screening^24^,^25^,^26^,^27^. The Griess assay for the determination of nitrite resulted from the reaction of NO with O2 was used as effective and efficient method to quantitate NO production^27^,^28^. Therefore, 18 compounds were primarily screened with a LPS stimulated RAW264.7 macrophage cell model for their anti-inflammatory activities evaluation. As shown in the results, most of compounds exhibited significant inhibition on nitrite production (Fig. 3A) suggesting the decreased production of NO. MTT results indicated that almost all of compounds exhibited no cytotoxicity except compound **10** (Figure S1) Among these compounds, the new compound **4** exhibited the highest inhibitory activity, far more than salvianolic acid B (Figure S2A), suggesting compound **4** was able to be a potential anti-inflammatory drug.

### Compound 4 Suppressed Activation of Pro-inflammatory Mediators in LPS-induced RAW264.7 Cells

Accumulated evidence demonstrated that pro-inflammatory stimuli activated various pro-inflammatory mediators including inducible iNOS (iNOS), COX-2, TNF-α, and IL-6 through NF-κB activation^29^,^30^. LPS stimulation induced high expression of iNOS in RAW264.7 cells^31^,^32^. COX-2, the key enzyme for biosynthesis of prostaglandins, also acts as an important player in inflammation^8^,^33^. Increased COX-2 expression is associated with inflammation, such as bronchitis, rheumatoid arthritis, *etc.*^4^. In this study, compound **4** could significantly decrease the nitrite content in LPS-stimulated RAW264.7 cells in a dose-response fashion suggesting the decreased generation of NO. A much higher potency than that of tanshinone IIA was also observed (Fig. 3B). This was further confirmed by the flow cytometry assay using DAF-FM staining, a specific NO fluorescent probe. Compound **4** could dramatically reversed LPS-induced shift of the fluorescent peak suggesting the decreased release of NO (Fig. 3C). The secretion of other pro-inflammatory mediators, TNF-α and IL-6, were also blocked sharply by compound **4** that was superior to positive drug salvianolic acid B (Figs 3D,E and S2B,C). In addition, compound **4** also showed an inhibitory effect on expression of the iNOS and COX-2 stimulated by LPS in a dose-dependent manner, which was stronger than positive drug salvainolic acid B (Figs 3F and S2D). Thus, the decreased NO production could be due to the inhibition of iNOS. To exclude the contribution of cell death to compound **4**’s inhibitory effect, a MTT assay was performed. Compound **4** had no obvious impact on the cell viability, even at the highest concentration (Figure S1). Taken together, these findings aforementioned corroborated that compound **4** displayed anti-inflammatory activity through inhibiting expression of iNOS and COX-2, as well as the secretion of pro-inflammatory mediators including NO, TNF-α, and IL-6 in LPS-stimulated RAW264.7 macrophages.

### Compound 4 Suppressed NF-κB Translocation in LPS-stimulated RAW264.7 Cells

**Figure 4:**
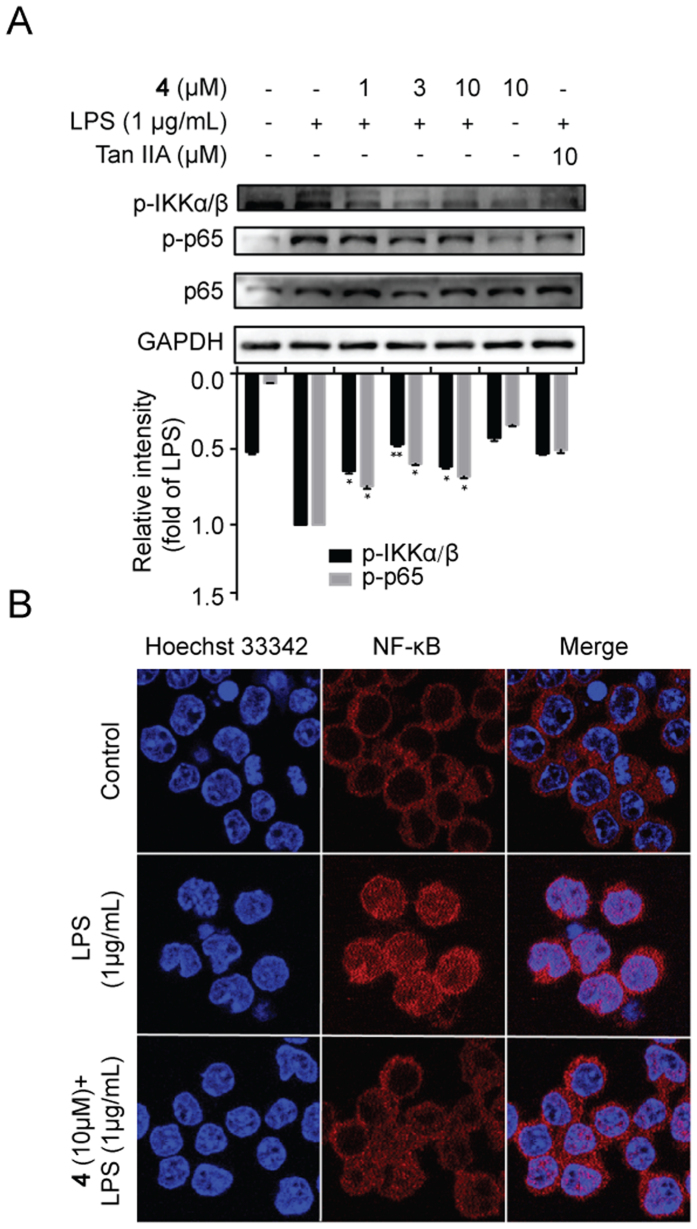
Compound 4 inhibited LPS-stimulated NF-κB activation. (**A**) Cells were pretreated with compound **4** as aforementioned. The protein expression of p-p65, p-65, and p-IKKα/β was determined by Western blotting. (**B**) Cells were pretreated with/without compound **4** (10 μM) for 1h and then treated with LPS (1 μg/mL) for another 1 h. The translocation of NF-κB p65 was examined by immunofluorescence. Tan IIA, tanshinone IIA.

The transcription factor NF-κB p65 plays pivotal roles in the control of pro-inflammatory genes expression. Dysregulation of NF-κB p65 has been medicated in the progression of many inflammatory diseases, tumors, immune dysfunctions, *etc.*^34^. Translocation from cytoplasm to nucleus, NF-κB p65 is indispensable for the activation of inflammatory genes expression and secretion, including COX-2, TNF-α, iNOS, IL-1b, IL-6 and IL-8^34^,^35^. Therefore, we explored the effect of compound **4** on expression and nuclear translocation of NF-κB p65. The expression of p-p65 induced by LPS was dose-dependently inhibited by compound **4** (Fig. 4A). Furthermore, immunofluorescence results showed that nuclear expression of p65 stimulated by LPS was significantly reversed after treatment with compound **4** (Fig. 4B) suggesting that compound **4** was able to disrupt translocation of p65 in LPS-induced RAW264.7 cells. In addition, compound **4** could markedly decrease the expression of the p-IKK-α/β (Fig. 4A). Collectively, these findings suggested that compound **4** suppressed LPS-induced NF-κB p65 activation and translocation.

### MAPKs Signaling Pathway Contributed to the Anti-inflammatory Activities of Compound 4

**Figure 5:**
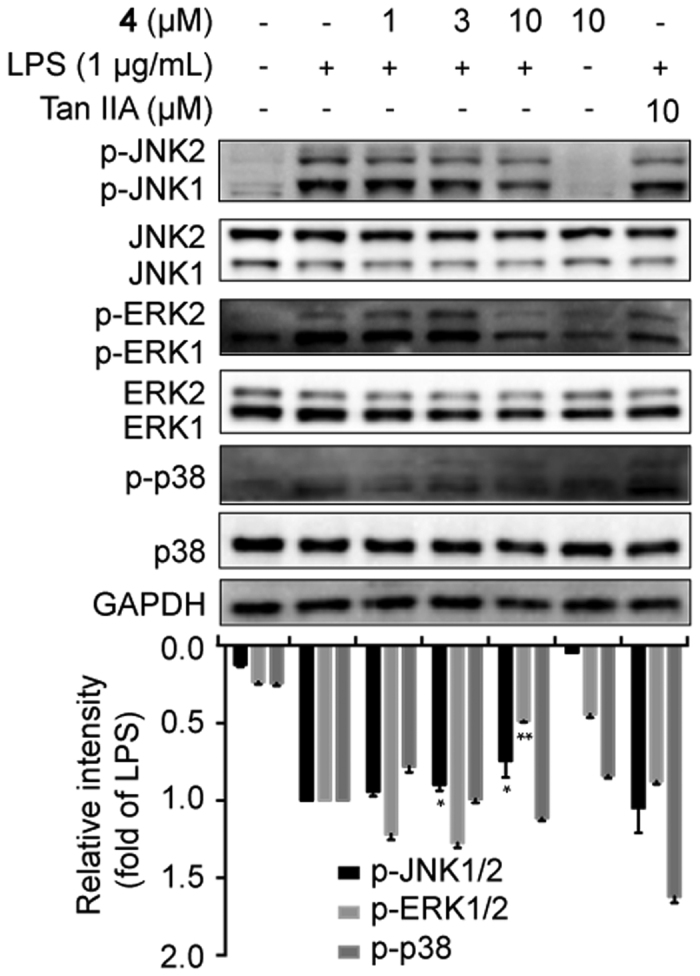
MAPKs contributed to the anti-inflammatory activity of compound 4. Cells were pretreated with compound **4** as aforementioned. The protein expression was determined by Western blotting. Tan IIA, tanshinone IIA.

The activation of MAPKs signaling including JNK1/2, ERK1/2, and p38MAPK was always involved in cellular responses to inflammatory stress^36^. To explore the role of JNK1/2, ERK1/2, or p38MAPK signaling in compound **4**’s anti-inflammatory effect, their protein expression was detected. As shown in Fig. 5, treated with compound **4**, the expression of p-JNK1/2 and p-ERK1/2 was dramatically decreased in a dose-dependent manner, while the expression of total JNK1/2 and ERK1/2 showed no changes. However, compound **4** demonstrated no obvious effect on p38MAPK. Collectively, these findings indicated that JNK and ERK might participate in the anti-inflammation process of compound **4** in LPS-stimulated RAW264.7 cells.

### Compound 4 Disrupted the TLR4 Dimerization Induced by LPS

**Figure 6:**
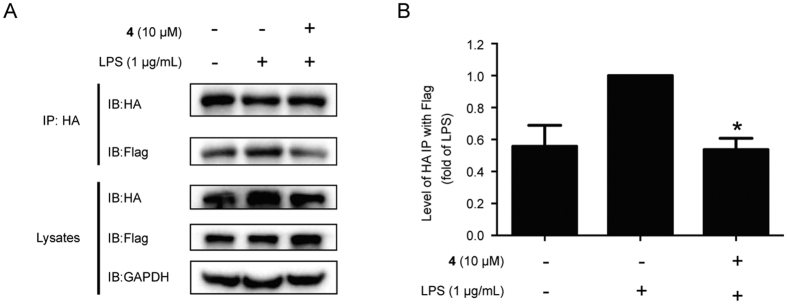
Compound 4 blocked LPS-induced TRL4 dimerization. (**A**) HEK293T cells transfected with TLR4-HA and TLR4-Flag were treated with compound **4** for 1 h. And then co-treated with LPS (1 μg/mL) for 24 h. Cells were collected and proteins were immuneprecipitated with anti-HA magnetic beads. Immunocomplexes were detected by western blotting with anti-HA and anti-Flag antibodies. (**B**) Quantification of TLR4-HA and TLR4-Flag in HEK293T cells. Quantification of TLR4-HA and TLR4-Flag was determined with densitometric analysis, and TLR4-Flag was compared to TLR4-HA. *p < 0.05 versus the LPS-treated group.

Based on the promising results of compound **4** at inhibiting NF-κB translocation and JNK1/2 and ERK1/2 actinvation *in vitro*, we further investigated whether toll-like receptor (TLR4) would be potentially modulated by compound **4**. TLR4, a transmembrane receptor located on surface of some of immune cells, is critical signaling receptor for LPS that mediates innate and acquired immunity^37^. Activated by LPS, TLR4 will be induced to dimer, and subsequently causes a MAPK signaling and nuclear translocation of transcription factors NF-κB through interacting between dimerized TLR4 and its downstream ‘bridging adaptor’ molecules, phosphorylated TRAM or MYD88, eventually triggering a pathogen-specific innate immune response by upregulating the proinflammatory cytokines release^38^,^39^. Therefore, to suppress the formation of homodimerization of TLR4 is proposed to be a new strategy to treat inflammatory diseases^40^. In this study, to detect the ability of compound **4** to disrupt TLR4 dimerization induced by LPS, we employed HEK293T cells co-transfected with TLR4-HA and TLR4-Flag plasmids. As shown in Fig. 6, the decreasing of TLR4-Flag in the TLR4-HA precipitation after treatment with compound **4** showed that compound **4** suppressed the TLR4 dimerization significantly compared to that of LPS group without compound **4** treatment. Therefore, we speculated that the potential anti-inflammatory effect of compound **4** may due to its inhibition on TLR4 dimerization.

### The Cytotoxicity of Isolated Compounds against Cancer Cells

**Figure 7:**
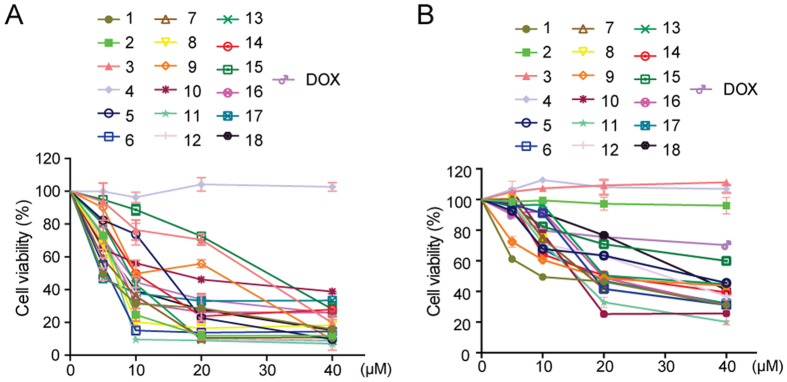
The cytotoxicity of the isolated compounds on cancer cells. H1299 cells (**A**) and Bel-7402 (**B**) were treated with compounds for 24 h and the cell viability was determined by MTT assay. DOX, doxorubicin.

Previous studies have shown that some tanshinones, such as tanshinone IIA, tanshinone I and cryptotanshinone exhibited anti-cancer properties both *in vitro* and *in vivo*^41^,^42^,^43^,^44^,^45^. However, the anti-cancer activities of some trace compounds such as **6, 7,** and **11** have not been tested. Herein, the toxicity of the isolated compounds towards non-small cells lung cancer H1299 cells and hepatocellular carcinoma Bel-7402 cells was tested. The IC_50_ values on H1299 cells and the inhibitory curves were shown in Table 3 and Fig. 7A, respectively. For most compounds, the IC_50_ values on human embryonic lung fibroblasts (HELF) were much higher than those on H1299 (Figure S3A). Structurally, the majority of isolated compounds belonged to diterpenes except compound **4**. In terms of the saturation level of A and D ring of isolated compounds, they could be divided into five categories. Compounds **1, 8** and **17** that have both saturated A and D ring belong to category A; compounds **10** and **15** that have both unsaturated A and D ring belong to category B; compounds **5**, **6**, **7**, **11**, **12**, **13** and **16** that have saturated A ring and unsaturated D ring belong to category C; compounds **19** and **14** that have unsaturated A ring and saturated D ring belong to category D, and the remnant compounds **2**, **3** and **4** belong to category E. From Table 2 and Fig. 7A, most compounds exhibited significant anti-cancer effect on H1299 bar compounds **2** and **4**. No cytotoxicity of compound **2** on H1299 cells indicated that an intact funan ring might be indispensable, which was consistent with previous report^46^. The cytotoxic activities of some compounds such as **1**, **6**, **7, 11**, **17**, **18**, *etc.* were higher than that of the positive control. Compounds from A category showed the most significant cytotoxicity against H1299 cells and compounds from C category demonstrated the second better activity followed by compounds from D category. Therefore, the anti-cancer activities of compounds were ranked as: A > C > D > B. Among these compounds from category C, compounds **6** and **7** showed better activities than that of others in the same category C, which indicated 3-OH played a vital role on the anti-cancer activity. In addition, the cytotoxicity of these compounds was less efficient against Bel-7402 than that of H1299 cells (Table 2 and Fig. 7B). Almost all the IC_50_ values were over 10 μM, even the IC_50_ value of the positive control doxorubicin was over 40 μM. The IC50 values of these compounds on LO2 cells were similar to those on Bel-7402 (Figure S3B), which indicated that they exhibited lower cytotoxicity to normal cells. However, the anti-cancer structure activity relationship (SAR) in Bel-7402 cells was similar to that of in H1299 cells. Taken together, SAR of these compounds in against both H1299 cells and Bel-7402 cells was as: A > C > D > B.

In summary, 18 compounds were separated and purified from Danshen and 4 of them were identified as new compounds. Most of them demonstrated anti-inflammation and anti-cancer activities. New compound **4** showed significant anti-inflammatory effect in LPS-induced RAW264.7 macrophages through suppressing the secretion of inflammatory cytokines and inhibiting the expression of iNOS and COX-2 possibly via the regulation of NF-κB as well as MAPKs pathways. In addition, blocking TLR4 dimerization might be the initial step for the anti-inflammatory effect of compound **4**. Finally, the cytotoxic effects of the isolated compounds on H1299 and Bel-7402 cells provide clues for the SAR for the first time.

### Experimental Process

The optical rotations were detected by a Perkin-Elmer model 241 polarimeter. IR spectra were recorded by a Perkin-Elmer 983 G spectrometer with KBr pellets. UV spectra data were determined by UV2401 spectrometer (Shimadzu Corp., Japan). NMR spectra were recorded on a Varian Inova 500 spectrometer. HRESIMS spectra were obtained on a Micromass Q-TOF2 spectrometer (Micromass Corp., UK). Silica gel (60–100 mesh, Qingdao Marine Chemical Ltd., People’s Republic of China) and Rever Agilent Zorbax SB-C18 semipreparative HPLC column (250 × 9.4 mm i.d., 5 μm, Agilent Corp. Palo Alto, CA, USA) on a Shimadzu HPLC system composed of a LC-20AT pump with a SPD-20A detector (Shimadzu Corp., Kyoto, Japan) were used for column chromatography (CC). Medium pressure liquid chromatography (MPLC) purification was performed on a Büchi Flash Chromatography system composed of a C-650 pump and a flash column (460 × 26 mm i.d., Büchi Corp., Flawil, Switzerland). Sephadex LH-20 was purchased from GE Corp (Piscataway, NJ, USA). Dulbecco’s modified Eagle’s minimum essential medium (DMEM), fetal bovine serum (FBS), were purchased from Life Technologies/Gibco Laboratories (Grand Island, NY, USA). 3-(4,5-Dimethylthiazol-2-yl)-2,5-diphenyl tetrazolium bromide (MTT), LPS (Escherichia coli, serotype 0111:B4), were purchased from sigma Sigma-Aldrich (St. Louis, MO, USA). Antibodies against iNOS, HA, Flag, COX-2, p-65 and p65 were purchased from Cell Signaling Technologies (Beverly, MA, USA).

### Plant Materials

Raw Danshen material was purchased from Bozhou (Anhui province, China) and identified by Prof. Xiao-ran Li. The raw danshen material was made the voucher specimen (No. DS2014-09-01),which was placed in the herbarium of the College of Pharmaceutical Science at Soochow University.

### Extraction and purification

The raw material (100 kg) was extracted twice with 95% EtOH under reflux. The solvent was removed under reduced pressure to yield condensed solution (50 L), which was subsequently re-dissolved in hot distilled water and successively extracted by dichloromethane (CH_2_Cl_2_) and n-butyl alcohol. CH_2_Cl_2_ fraction (5.2 kg) was further vacuum chromatographed on a silica gel (60–100 mesh) column (120 × 20 cm i.d.), eluted with a gradient of Petroleum ether-EtOAc (9:1, 6:1, 3:1, 1:1, 0:1). The combined eluate (3:1 and 1:1) (536.2 g) was then separated by MPLC over a C-18 column (110 × 12 cm i.d.) with MeOH-H_2_O (30:70, 40:60, 50:50, 60:40, 70:30, 80:20, 90:10, 100:0, each 5000 mL) to afford 8 fractions (1 to 8). Fraction 1 and Fraction 2 were combined to Sephadex LH-20 gel column chromatography (130 × 3 cm i.d.) with pure MeOH and then subjected to semi-preparative RP-HPLC with MeOH-H_2_O (70:30 to 75:25) to yield **2** (5.2 mg), **3** (3.6 mg), **5**(86.2 mg), and **6**(175.2 mg), **7** (32.2 mg). Fraction 3 was separated by semi-preparative RP-HPLC with MeOH-H_2_O (75:25 to 79:21) to furnish **1** (5.6 mg), **8** (16.8 mg), **9** (6.2 mg), and **10** (22.5 mg). Fraction 4 was performed on semi-preparative RP-HPLC with MeOH-H_2_O (75:25 to 81:19) to obtain **4** (3.1 mg), **11** (4.2 mg), and **12** (7.8 mg). Fraction 5 was performed on semi-preparative RP-HPLC with MeOH-H_2_O (78:22 to 85:15) to give **4** (41.2 mg) and **13** (6.2 mg). Fraction 6 was performed on semi-preparative RP-HPLC with MeOH-H_2_O (82:18 to 90:10) to obtain **14** (5.7 mg), **15** (7.5 mg), **16** (2.1 mg), **17** (3.4 mg), and **18** (4.2 mg).

Compound **1**(tanshinol C): red powder; 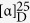 -132.1 (c = 0.13, MeOH); UV (MeOH) *λ*_max_ (log *ε*): 220 (4.02), 266 (3.75) nm; IR(KBr) *ν*_max_: 3368, 2967, 1637, 1640, 1255, 912 cm^−1^; ^1^H-NMR (C_5_D_5_N, 500MHz) and ^13^C-NMR (C_5_D_5_N, 125MHz) spectroscopic data see Table 1; HRESIMS (positive ion mode) m/z: 299.1275 [M + H]^+^ (calcd for C_18_H_19_O_4_, 299.1275).

Compound **2** (tanshinol D): orange powder; 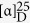 -124.1 (c = 0.12, MeOH); UV (MeOH) *λ*_max_ (log *ε*): 214 (3.82), 261 (4.15), 293 (3.25) nm; IR(KBr) *ν*_max_: 3379, 2974, 1638, 1460, 1285, 835 cm^−1^; ^1^H-NMR (CD_3_OD, 500MHz) and ^13^C-NMR (CD_3_OD, 125MHz) spectroscopic data see Table 1; HRESIMS (positive ion mode) m/z: 333.1336 [M + H]^+^ (calcd for C_18_H_21_O_6_, 333.1336).

Compound **3** (tanphenol A): orange red powder; 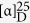 -134.3(c = 0.09, MeOH); UV (MeOH) *λ*_max_ (log *ε*): 220 (4.03), 257 (3.56), 278 (4.12) nm; IR(KBr) *ν*_max_: 3392, 2976, 1637, 1176, 934 cm^−1^; ^1^H-NMR (C_5_D_5_N, 500MHz) and ^13^C-NMR (C_5_D_5_N, 125MHz) spectroscopic data see Table 1; HRESIMS (positive ion mode) m/z: 315.1579 [M + H]^+^ (calcd for C_19_H_23_O_4_, 315.1579).

Compound **4** (diethyl blechnic): yellow powder; 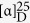 -36 (c = 0.06, MeOH); UV (MeOH) *λ*_max_ (log *ε*): 221 (3.12), 257 (4.17), 278 (4.12) nm; IR(KBr) *ν*_max_: 3300, 2600, 1720, 1690, 1610 cm^−1^; ^1^H-NMR (C_5_D_5_N, 500MHz) and ^13^C-NMR (C_5_D_5_N, 125MHz) spectroscopic data see Table 1; HRESIMS (positive ion mode) m/z: 415.1389 [M + H]^+^ (calcd for C_22_H_23_O_8_, 415.1389).

### Cell Culture

Cell lines H1299, HEK293T and Bel-7402 were obtained from American Type Culture Collection (ATCC, Manassas, VA, USA). HELF, LO2, and RAW264.7 macrophage cell line obtained from Cell Bank of the Chinese Academy of Sciences (Shanghai, China). HEK293T, HELF and RAW264.7 cells were grown in DMEM with 10% FBS and H1299, Bel-7402 and LO2 were cultivated in RPMI1640 with 10% FBS. Cell cultures were maintained at 37 °C under a humidified atmosphere of 5% CO_2_ in an incubator.

### MTT Assay for Cell Viability

H1299 and Bel-7402 cells were seeded a density of 10^5^ cells/mL in 96-well plates, and RAW264.7 cells were at a density of 10^6 ^cells/mL. Cells were subsequently exposed to compounds at indicated concentrations for 24 h. After treatment, 0.5 mg/mL MTT was then added to the wells and the plates were incubated for further 3 h. Subsequently, 100 μL of DMSO was then added to each well to dissolve the crystals. The absorbance was recorded at the wavelength of 570 nm in Benchtop Multi-Mode Microplate Reader (Molecular Devices, Sunnyvale, CA).

### Determination of Nitrite

Plated into 24-well plates at the density of 5 × 10^5^ per well, RAW264.7 cells were treated in absence or presence of indicated compounds at different concentrations and stimulated with or without LPS (1 μg/mL) overnight. The Griess reagent was used to measure and quantitate nitrite levels in culture media by a standard sodium nitrite curve followed by the instruction from manufacturer. The absorbance was read out in microplate reader at the wavelength of 540 nm.

### Determination of NO

Treated with compounds in the same conditions as mentioned above, at least 1 × 10^4^ cells were collected and labeled with DAF-FM diacetate (3 μM) for further 1 h at 37 °C. The fluorescence signal of each sample was the analyzed by a FACScanTM flow cytometer and determined by the FITC channel.

### ELISA assay for TNF-α and IL-6 levels

Pretreated with compound **4** for 1 h, RAW264.7 cells were then incubated with LPS at the concentration of 1 μg/mL for 18 h. The secretion of TNF-α and IL-6 from cells to culture media were determined by ELISA kits (Neobioscience, Shenzhen, China) following manufacturers’ instructions.

### Immunofluorescence

The immunofluorescence analysis of NF-κB p65 was performed as previously described^34^. Briefly, 2 × 10^5^ RAW264.7 macrophages were plated into a 35-mm glass-bottom SPL confocal dish and cultured overnight. Exposed to compound **4** for 1 h followed by simulation with LPS for another 1 h, cells were fixed and stained with DAPI for imaging. The imaging was performed under a Leica TCS SP8 laser confocal microscope using the 63X magnification with excitation/emission wavelength at 588/615–690 nm.

### Transfection with TLR4 Plasmids

HEK 293T cells were seeded into a dish (10 cm i.d.) at density of 10^6^ cells/dish for overnight. TLR4-HA and TLR-Flag (a gift from Naixin Kang, Soochow University) were transfected with Tubofect Transfection Reagents (Thermo Fisher Scientific, USA) for 24 h. Then cells were trypsinized and seeded into a dish (5 cm i.d.) at density of 5 × 10^5^ for overnight. Cells treated with indicated compounds were harvested in 24 h.

### Immunoblotting and Immunoprecipitation

Protein samples were extracted from the cells treated compound **4** or LPS and the concentration of samples were determined with a Pierce Biotechnology BCA protein assay kit. Then, 800 μg proteins in IP lysis buffer was immumoprecipiated with anti-HA magnetic beads (Pierce Biotechnology, Rockford, IL, USA) for 1 h at room temperature. The magnetic beads in 1% SDS loading buffer were boiled at 95–100 °C for 10 minutes. Equivalent amounts of each protein sample was subjected to SDS-PAGE gel electrophoresis and transferred from the gel to PVDF membranes. Blocked in 5% nonfat milk dissolved in Tris-buffered saline -Tween 20 (TBST) buffer for 1 h, the membranes were probed with primary antibodies at 1:1000 dilution in TBST buffer overnight at 4 °C and specific secondary antibodies at 1:5000 dilution in TBST buffer for further 1 h at 25 °C. The signal of protein bands were detected using SuperSignal West Femto Maximum Sensitivity Substrate (Pierce Biotechnology) under visualization in ChemiDoc™ MP Imaging System (Bio-Rad, Hercules, CA, USA).

### Data analysis

All results were presented as means ± SD. For statistical analysis, the significance of the intergroup differences was analyzed with the one-way analysis of variance (one-way-ANOVA) using GraphPad Prism 6.0 software. The significant difference was defined as *p* < 0.05.

## Additional Information

**How to cite this article**: Gao, H. *et al*. Tanshinones and diethyl blechnics with anti-inflammatory and anti-cancer activities from *Salvia miltiorrhiza* Bunge (Danshen). *Sci. Rep.* **6**, 33720; doi: 10.1038/srep33720 (2016).

## Supplementary Material

Supplementary Information

## Figures and Tables

**Figure 1:**
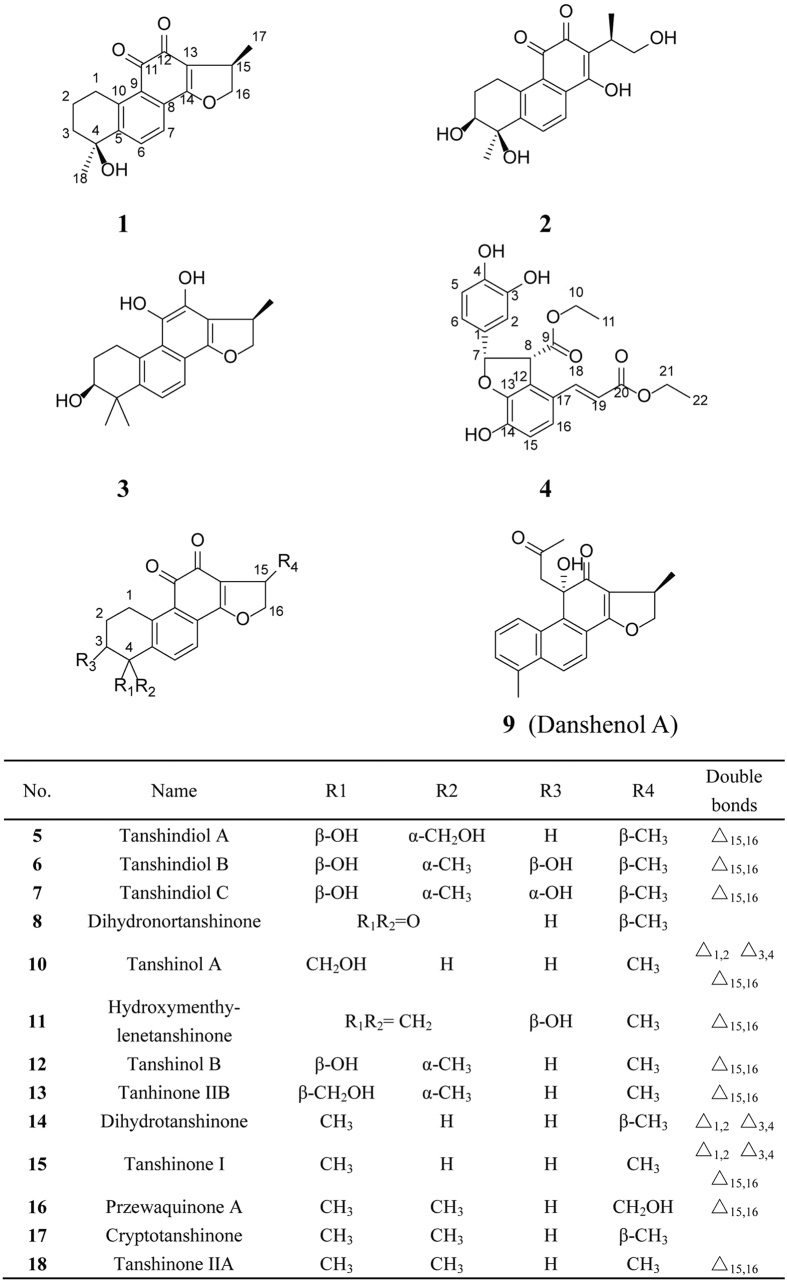
Structures of compounds 1–18.

**Table 1:** ^1^H and ^13^C NMR Data for Compounds 1–2.

Position	**1**		**2**	
	*δ*_C_	*δ*_H_	*δ*_C_	*δ*_H_
1	20.9	1.94 (2H, m)	25.3	3.30 (2H, m)
2	29.8	3.20, 3.36 (2H, m)	27.0	2.02 (2H, m)
3	39.2	2.03, 2.12 (2H, m)	73.0	3.83 (1H, m)
4	70.4		73.1	
5	151.9		150.2	
6	133.7	8.32 (1H, d, 7.5)	134.7	8.03 (1H, d, 8.0)
7	123.5	7.65 (1H, d, 7.5)	124.2	7.64 (1H, d, 8.0)
8	128.2		128.4	
9	129.1		128.7	
10	142.9		143.7	
11	184.9		185.1	
12	176.6		176.8	
13	119.2		119.7	
14	168.6		173.3	
15	35.6	3.52 (1H, m)	35.7	3.56 (1H, m)
16	81.9	4.20, 4.76 (2H, m)	83.2	4.97,4.37 (2H, m)
17	18.9	1.29 (3H, d, 6.8)	18.9	1.33 (3H, d, 6.8)
18	32.5	1.69 (1.69, 3H, s)	29.6	1.45 (3H, s)

**Table 2:** ^1^H and ^13^C NMR Data for Compounds 3–4.

Position	**3**		**4**	
	*δ*_C_	*δ*_H_	*δ*_C_	*δ*_H_
1	37.1	1.69 (2H, m)	132.8	
2	26.8	1.45, 2.19 (2H, m)	114.0	7.44 (1H, d, 2.1)
3	77.9	5.21 (1H, dd, 11.7, 5.2)	147.8	
4	34.8		148.0	
5	144.7		116.8	7.22 (1H, d, 7.4 Hz)
6	130.3	7.63 (1H, d, 7.8)	117.8	7.04 (1H, dd, 8.1, 2.0)
7	132.1	7.97 (1H, d, 7.8)	87.9	6.46 (1H, d 4.7)
8	129.4		56.8	4.91 (1H, 4.7)
9	124.1		172.2	
10	148.2		62.0	4.20 (2H, m)
11	167.2		14.2	1.12 (3H, t, 5.1)
12	165.7		123.6	
13	114.1		148.9	
14	160.7		145.8	
15	39.5	3.70 (1H, m)	118.8	7.18 (1H, d, 8.4)
16	78.5	4.36 (1H, dd, 8.9, 4.3) 4.81 (1H, dt, 9.1)	121.3	7.40 (1H, d, 8.4)
17	30.6	1.32 (3H, s)	126.7	
18	31.4	1.10 (3H, s)	142.3	8.21 (1H, d, 15.9)
19	20.8	1.61 (3H, d, 6.7)	117.0	6.62(1H, d, 15.9)
20			167.3	
21			60.4	4.27 (2H, m)
22			14.6	1.21 (3H, t, 5.1)

**Table 3:** The IC_50_ values of all isolated compounds on H1299 and Bel-7402, respectively.

Compound	H1299 IC_50_ (μM)	Bel IC_50_ (μM)	Compound	H1299 IC_50_ (μM)	Bel IC_50_ (μM)
1	4.42 ± 0.21	11.33 ± 0.39	**11**	6.25 ± 0.47	15.82 ± 1.32
2	7.05 ± 0.26	NE	**12**	7.61 ± 0.37	27.02 ± 1.84
3	24.3 ± 0.34	NE	**13**	8.87 ± 0.47	29.20 ± 2.56
4	NE	NE	**14**	11.2 ± 0.62	22.93 ± 0.91
5	13.42 ± 1.24	32.31 ± 1.83	**15**	28.06 ± 1.52	NE
6	5.11 ± 0.36	21.45 ± 2.10	**16**	8.29 ± 1.01	23.27 ± 0.56
7	1.13 ± 0.21	19.81 ± 1.56	**17**	2.35 ± 0.15	19.97 ± 0.45
8	6.52 ± 0.27	19.81 ± 0.82	**18**	8.21 ± 0.24	34.07 ± 0.95
9	14.96 ± 0.68	22.11 ± 0.62	DOX	10.26 ± 0.37	≥ 40
10	15.93 ± 0.83	19.46 ± 1.27			

All experiments were repeated three times. NE indicated no effect and doxorubicin (DOX) was used as positive control.
