# Supplementary material for: Tanshinones and diethyl blechnics with anti-inflammatory and anti-cancer activities from Salvia miltiorrhiza Bunge (Danshen)
Source: Sci Rep. 2016 Sep 26;6:33720. doi: 10.1038/srep33720 (PMC5036060; doi:10.1038/srep33720)
Supplement: Supplementary Information [file srep33720-s1.pdf]

Tanshinones and diethyl blechnics with anti-inflammatory and anti-cancer activities from *Salvia miltiorrhiza* Bunge (Danshen)

Hongwei Gao, Wen Sun, Jianping Zhao, Xiaxia Wu, Jin-Jian Lu, Xiuping Chen, Qiong-ming Xu, Ikhlas A. Khan, Shilin Yang

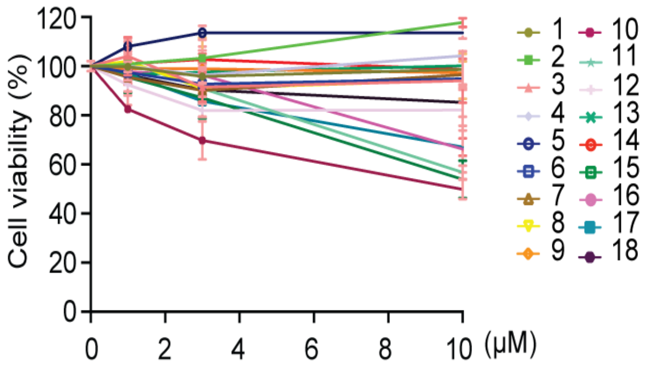

Figure S1. The cytotoxicity of compounds (1-18) on RAW264.7 cells. RAW264.7 cells were treated with these compounds for 24 h and the cell viability was determined by MTT assay.

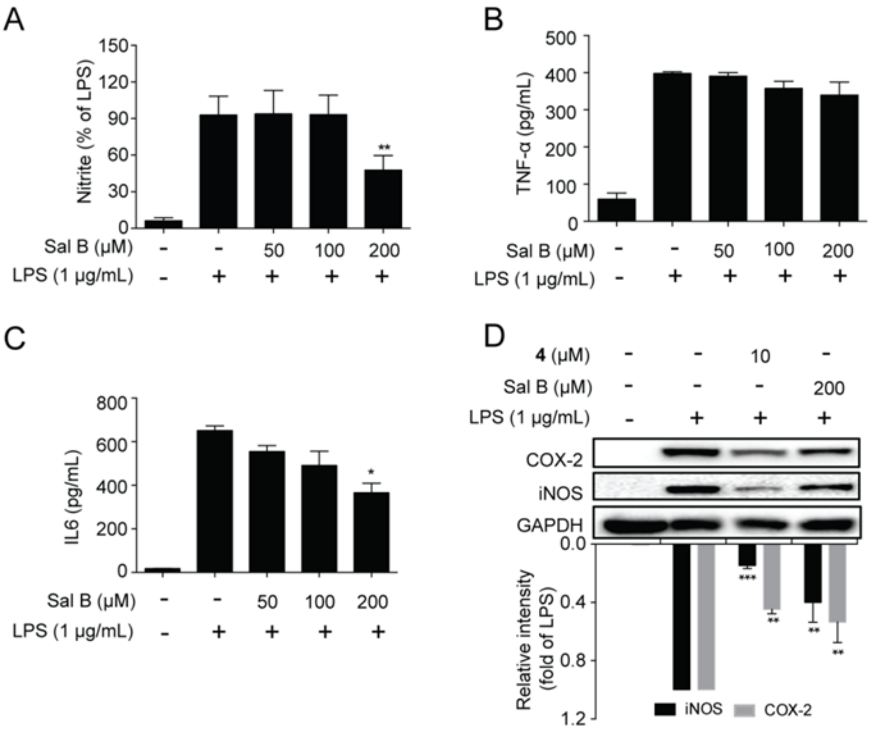

Figure S2. Anti-inflammatory effect of salvianolic acid B and compound **4.** (A) Cells were pretreated with salvianolic acid B for 1 h and then stimulated with LPS (1 μg/mL) for 24 h. The nitrite production was determined by Griess assay. $^{*}p< 0.05$, $^{**}p< 0.01$ and $^{***}p < 0.001$ versus LPS-treated group. (B, C) Cells were pretreated with

salvianolic acid B for 1 h and then stimulated with LPS (1 μg/mL) for 24 h. The levels of TNF-α and IL-6 in the culture medium were determined by ELISA assay. $^{*}p<0.05$ versus the LPS-induced group. (D) Cells were pretreated with salvianolic acid B or compound **4** for 1 h and then stimulated with LPS (1 μg/mL) for 24 h. The protein expression of iNOS and COX-2 was determined by Western blotting.

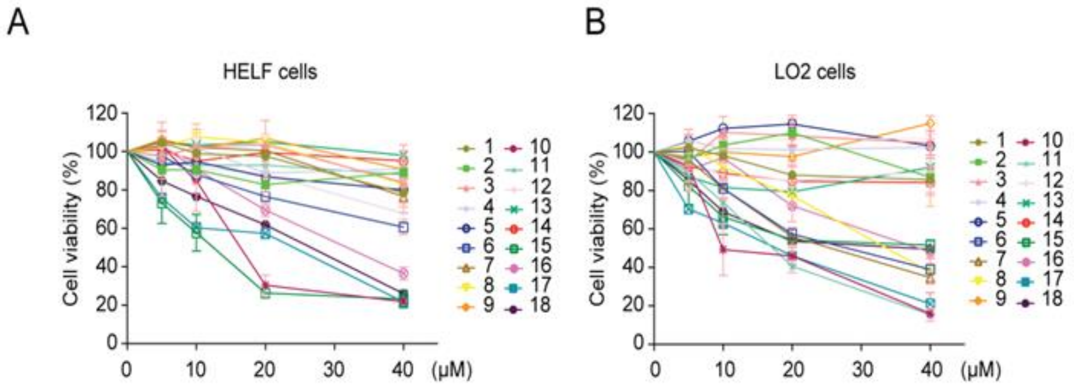

Figure S3. The cytotoxicity of compounds (1-18) on HELF and LO2 cells. HELF and LO2 cells were treated with these compounds for 24 h and the cell viability was determined by MTT assay.

Supporting Information

Figure s3-s10. HRESIMS and NMR spectra of tanshinol C(**1**)

Figure s11-s17. HRESIMS and NMR spectra of tanshinol D (**2**)

Figure s18-s24. HRESIMS and NMR spectra of tanphenol A (**3**)

Figure s25-s31. HRESIMS and NMR spectra of diethyl blechnic (**4**)

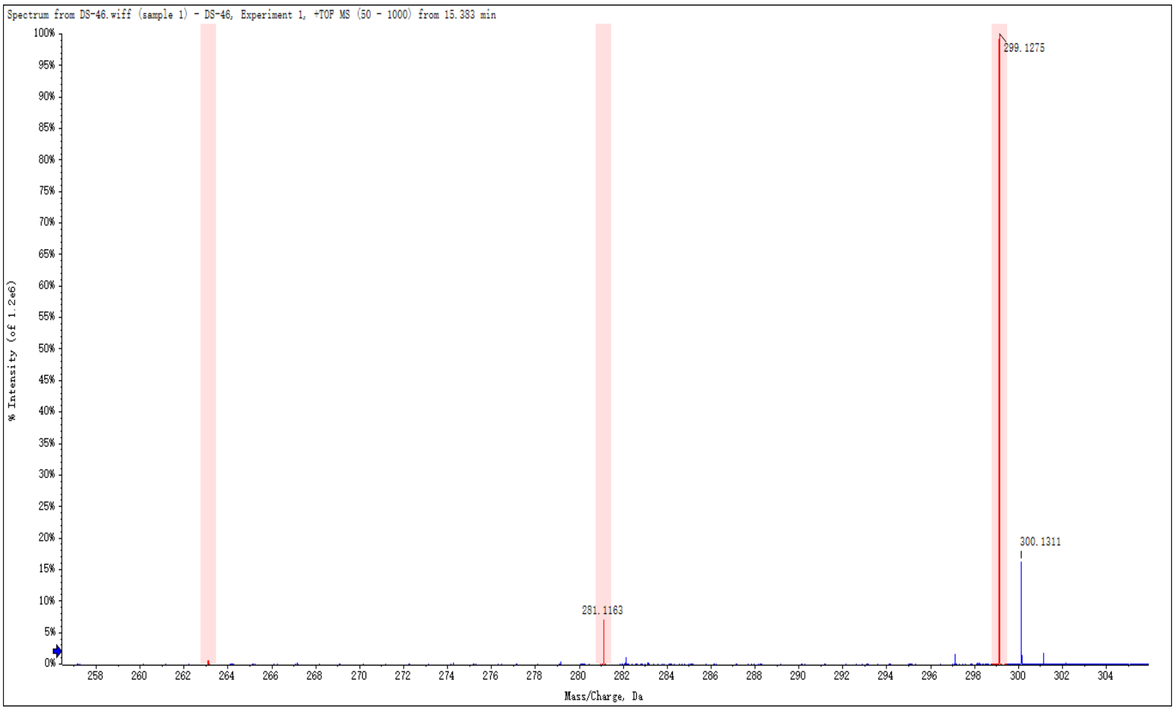

Figure s4 HRESIMS of tanshinol C (**1**)

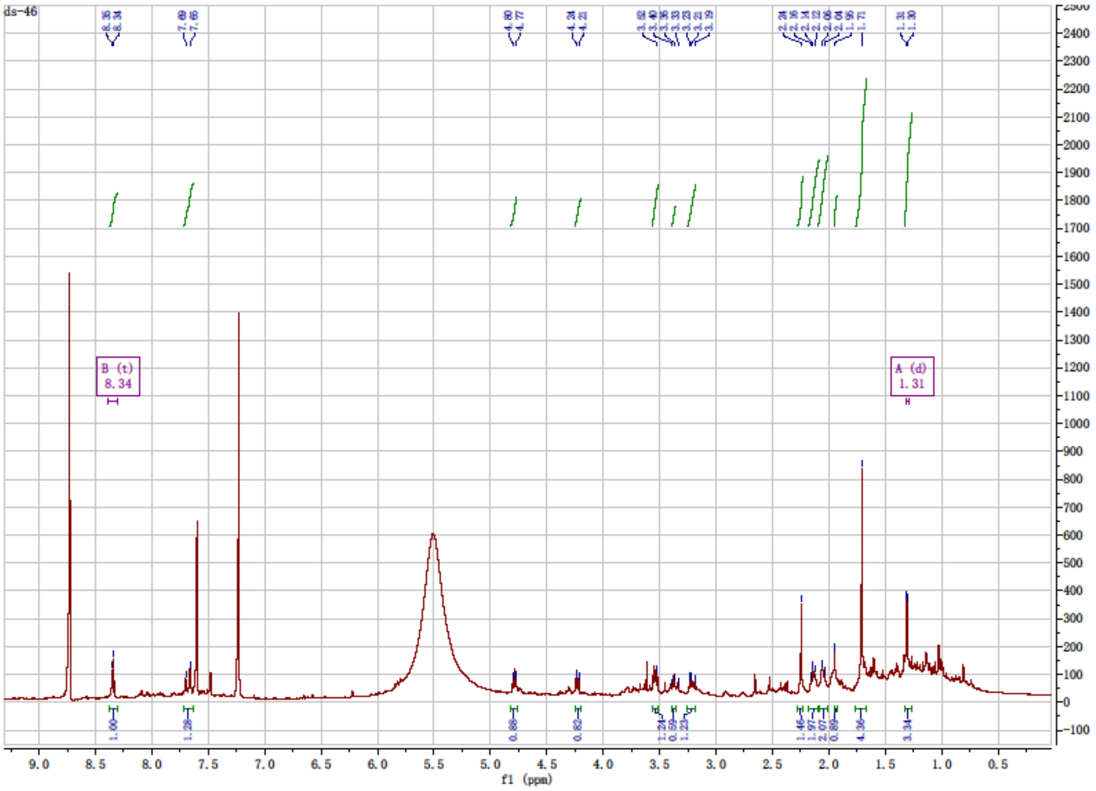

Figure s5 ^{1}H NMR of tanshinol C (**1**)

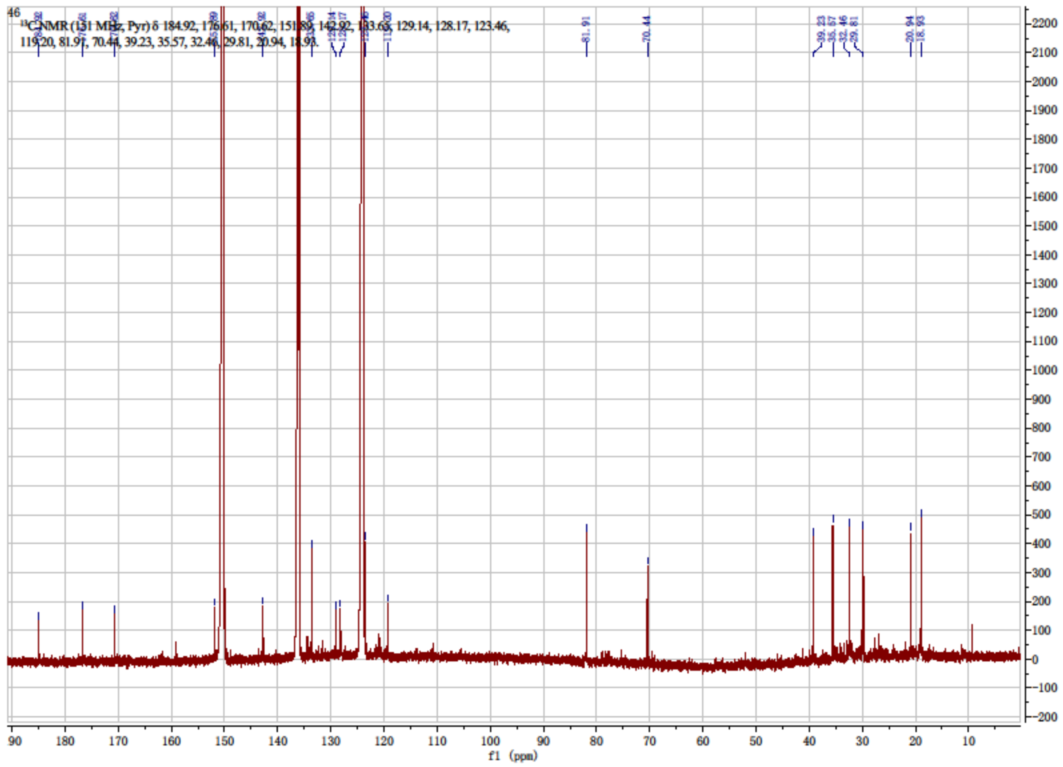

Figure s6 ^{13}C NMR of tanshinol C (**1**)

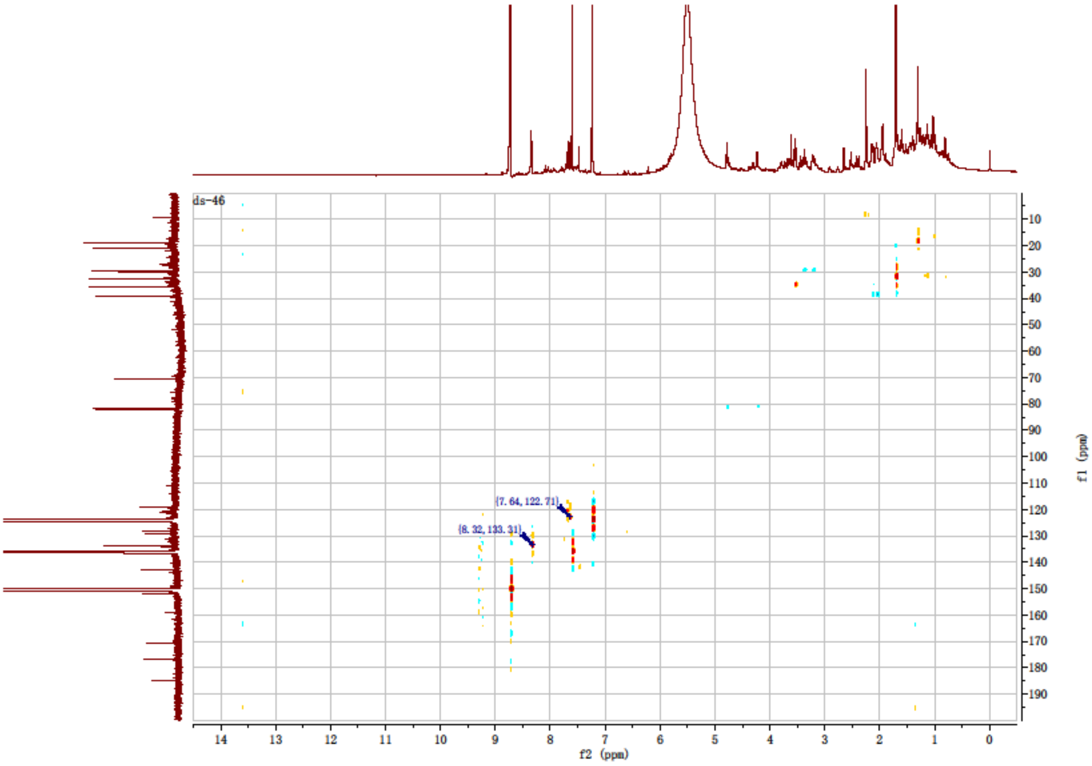

Figure s7 HSQC of tanshinol C (**1**)

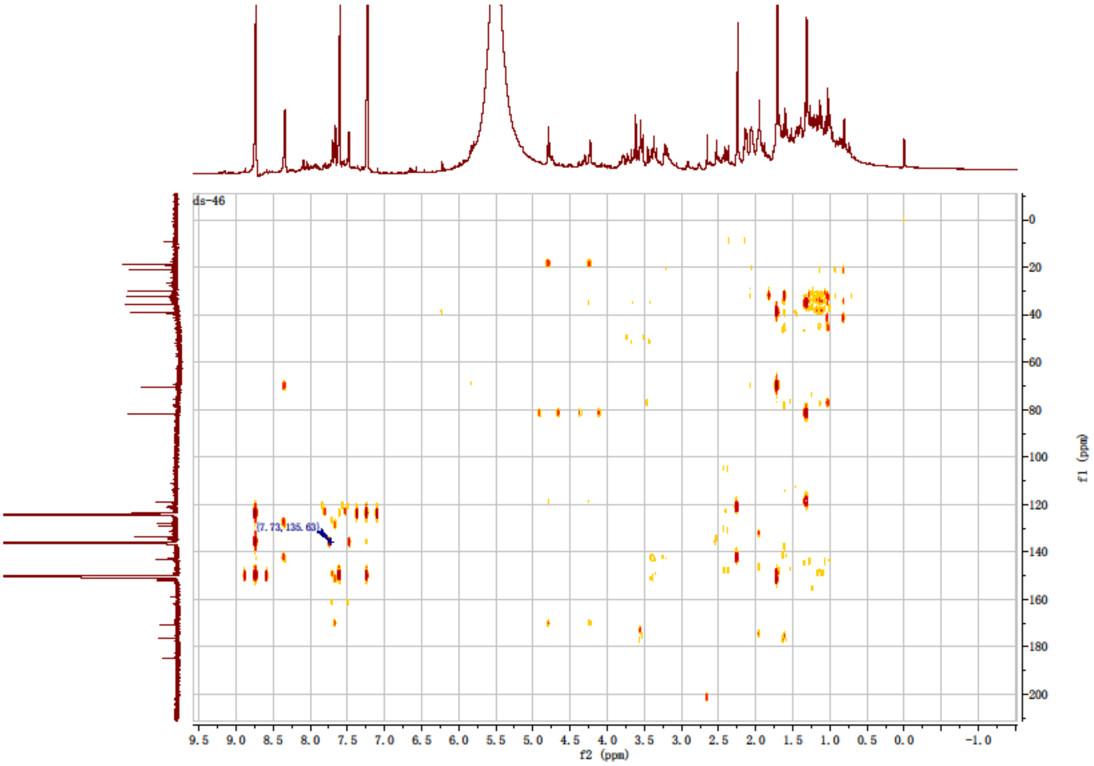

Figure s8 HMBC of tanshinol C (**1**)

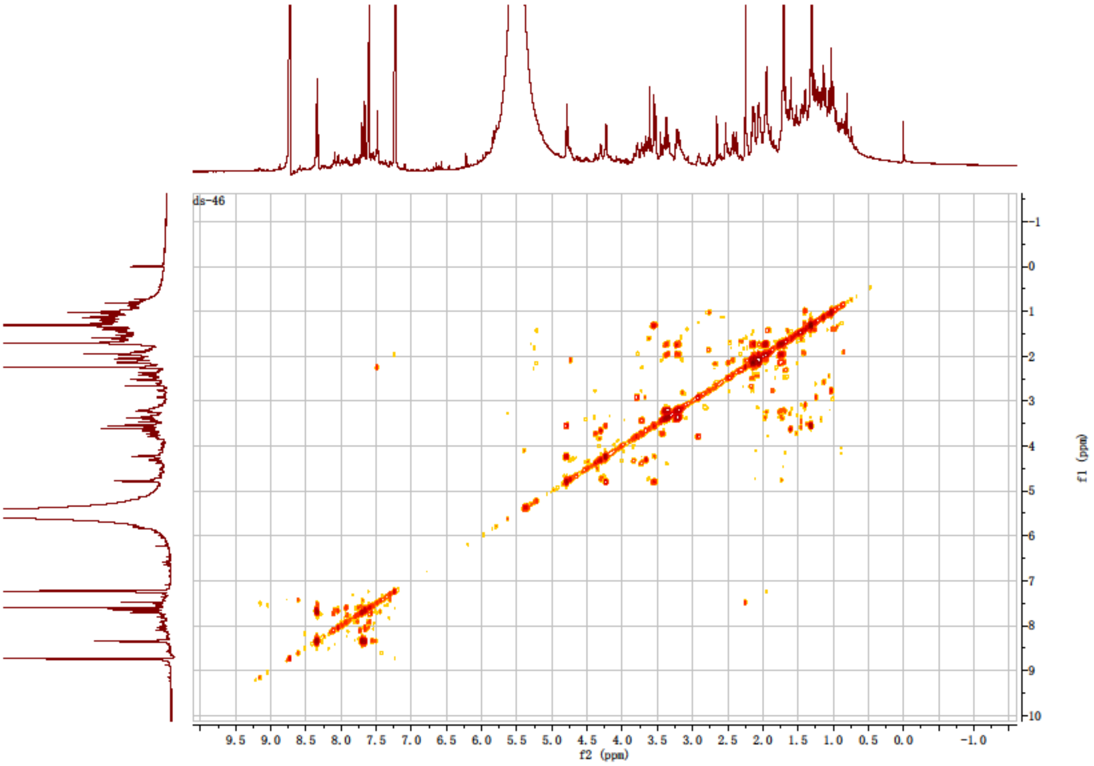

Figure s9 COSY of tanshinol C (**1**)

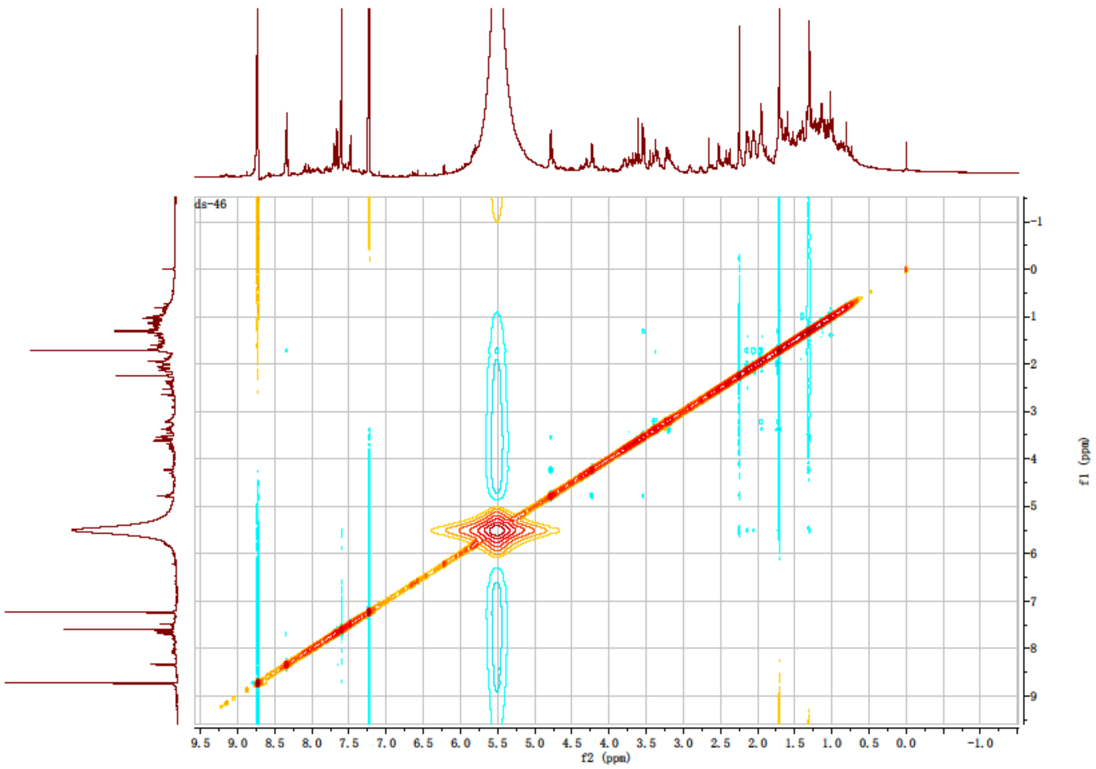

Figure s10 NOESY of tanshinol C (**1**)

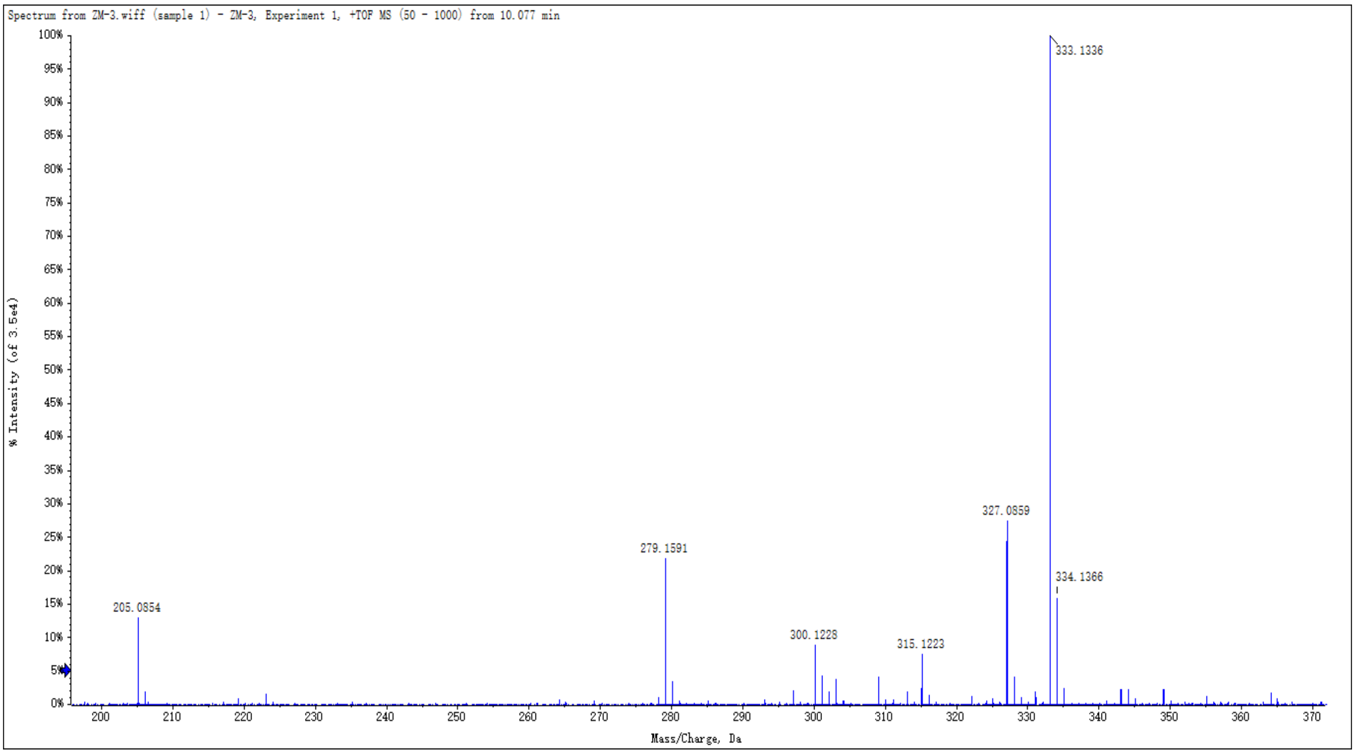

Figure s11 HRESIMS of tanshinol D (**2**)

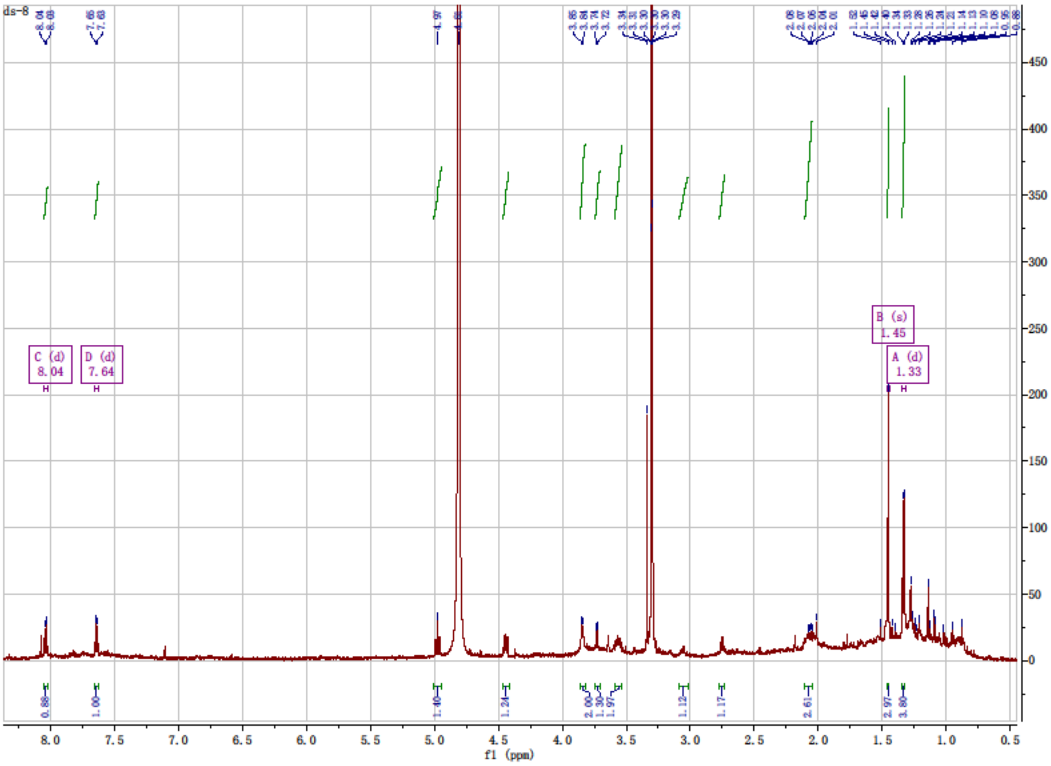

Figure s12 ^{1}H of tanshinol D (**2**)

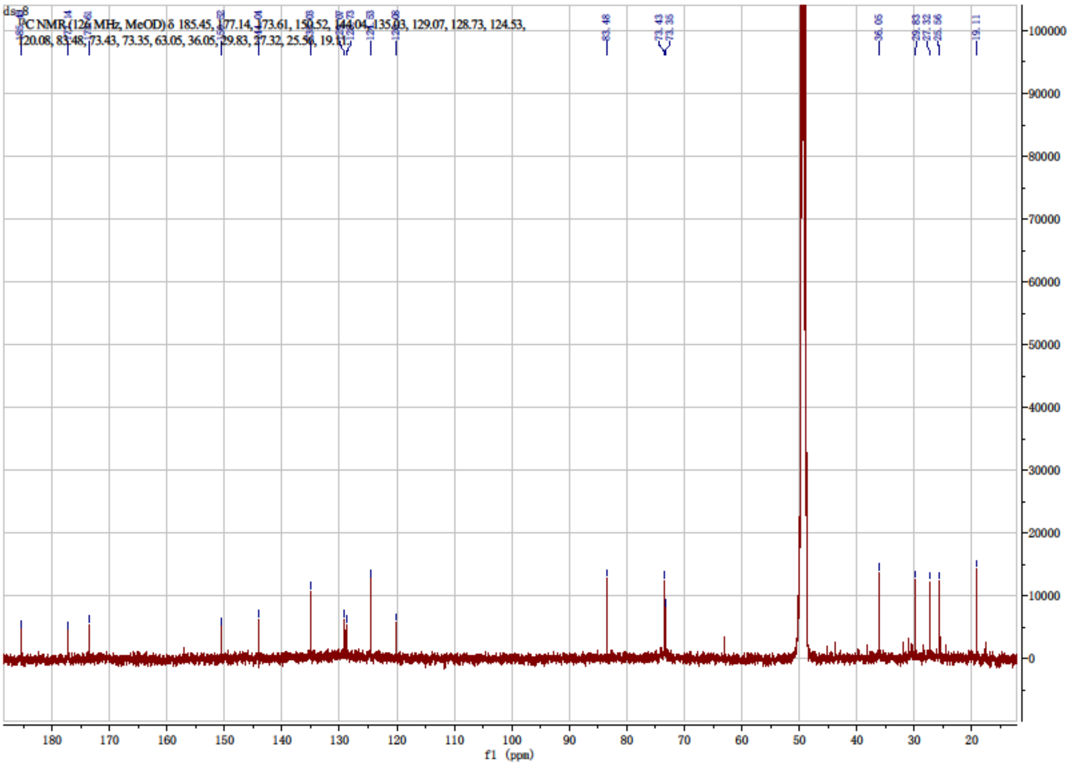

Figure s13 ^{13}C of tanshinol D (**2**)

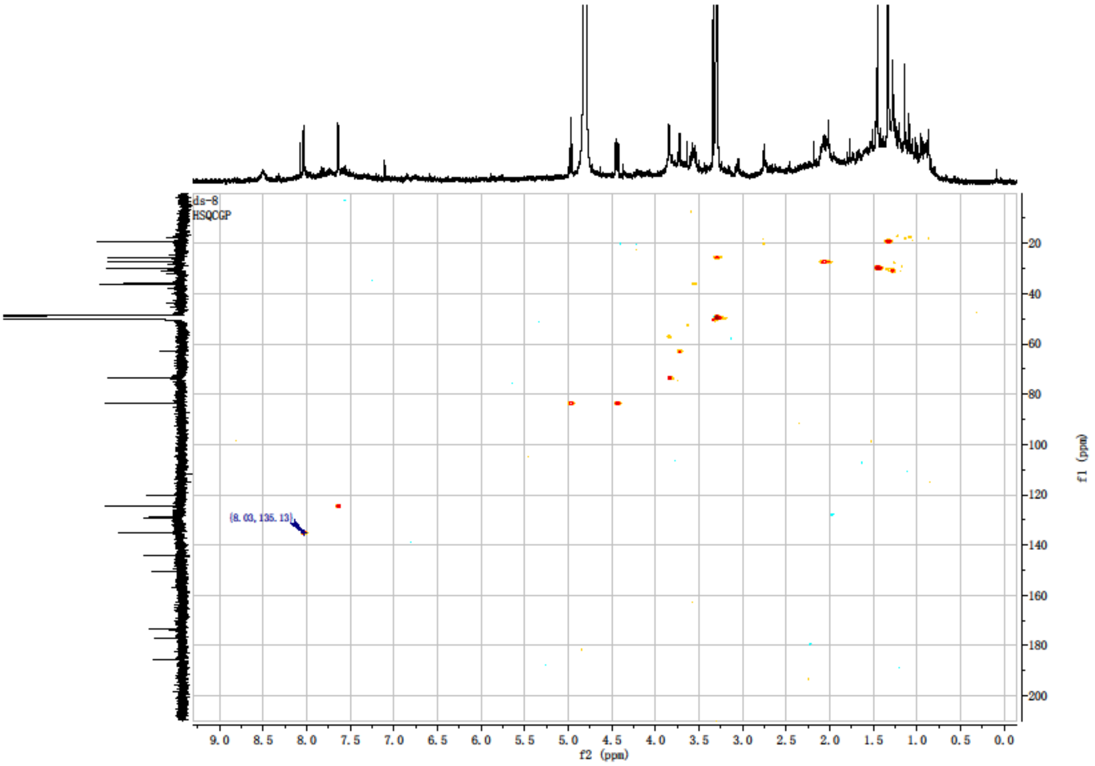

Figure s14 HSQC of tanshinol D (**2**)

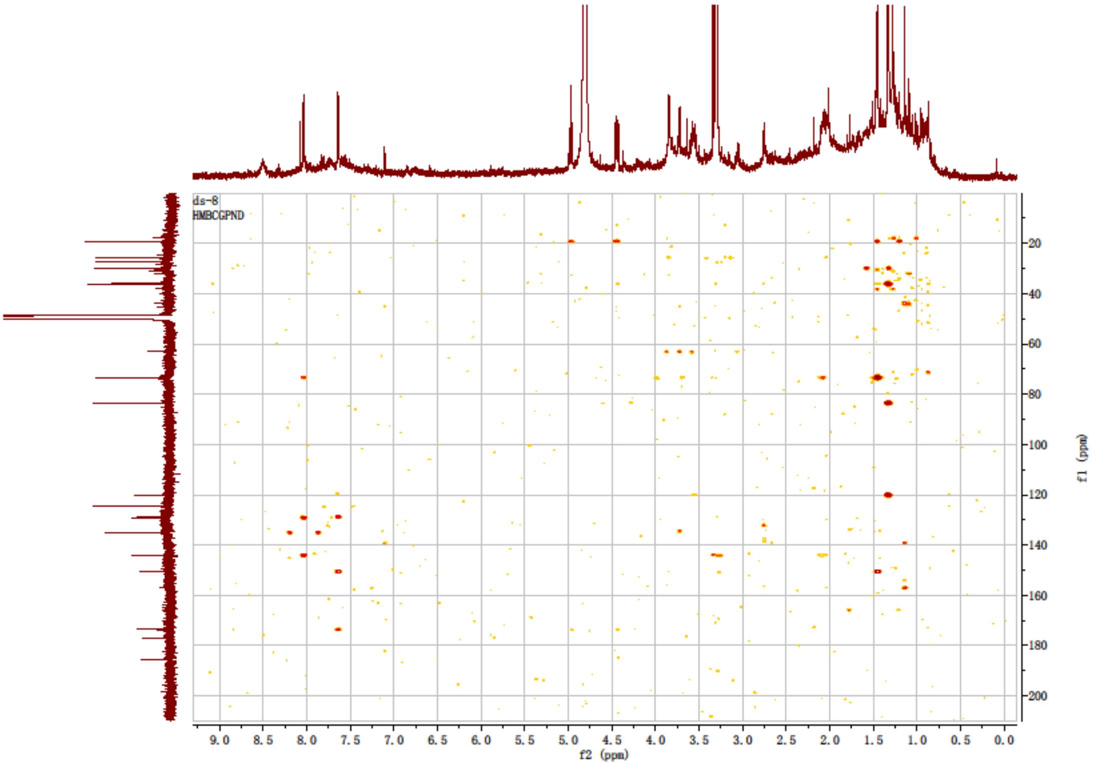

Figure s15 HMBC of tanshinol D (**2**)

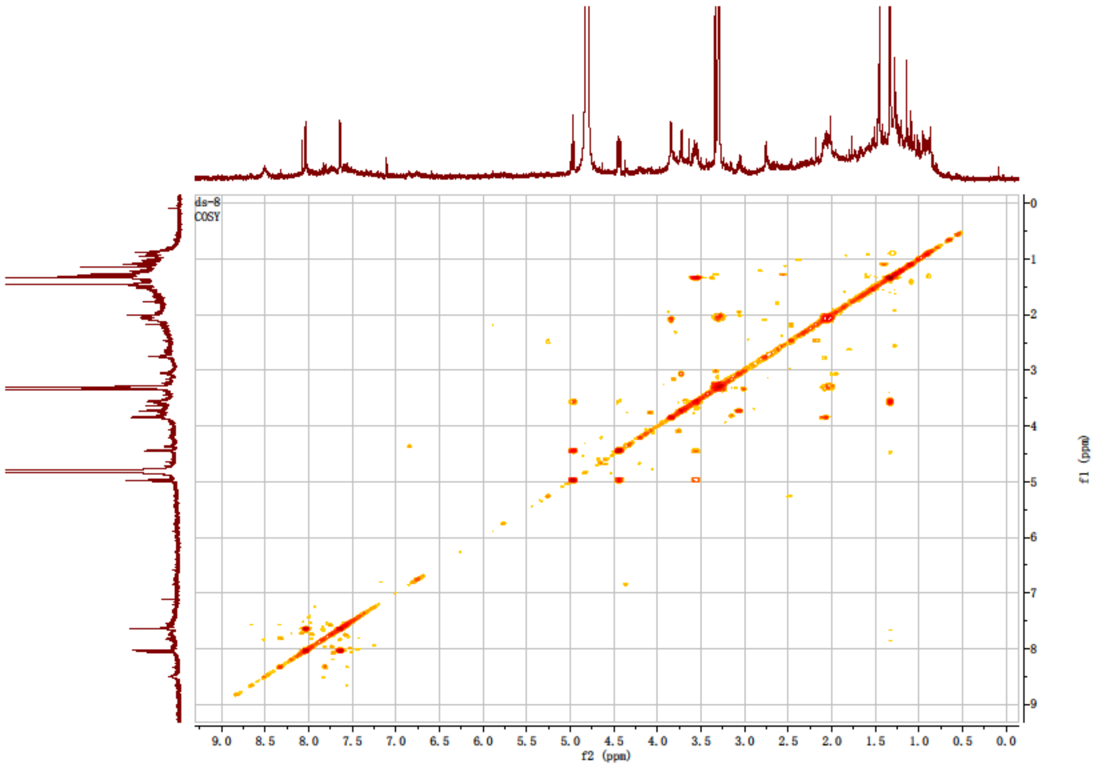

Figure s16 COSY of tanshinol D (**2**)

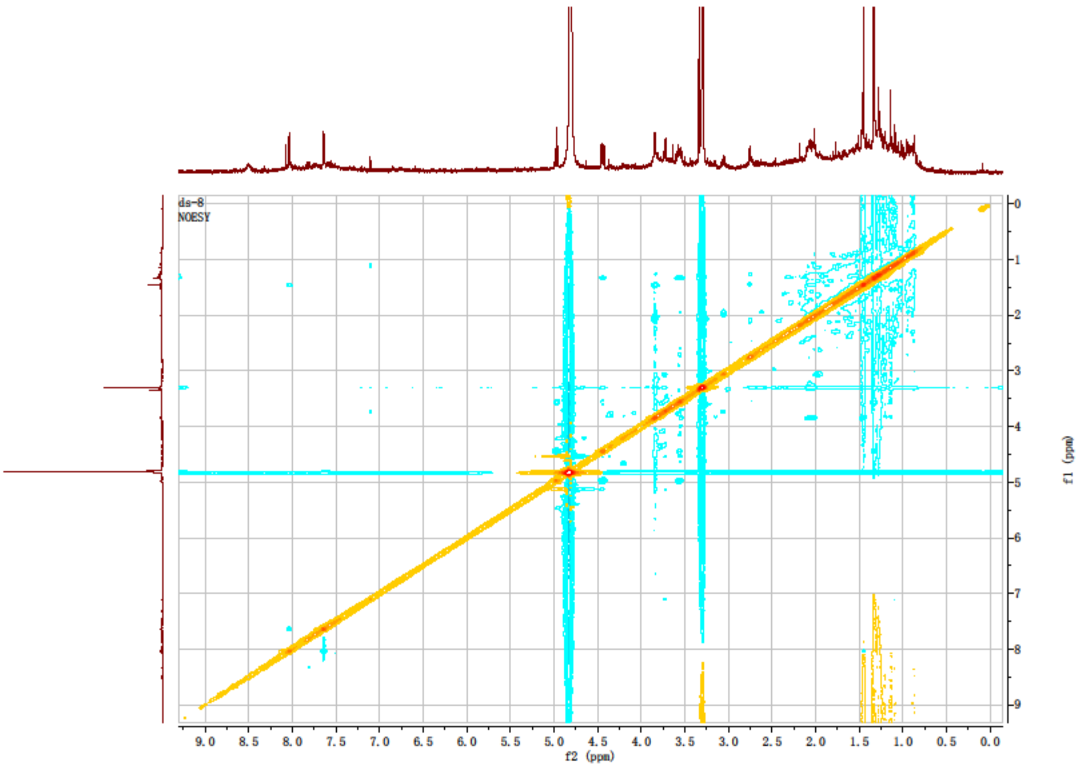

Figure s17 NOESY of tanshinol D (**2**)

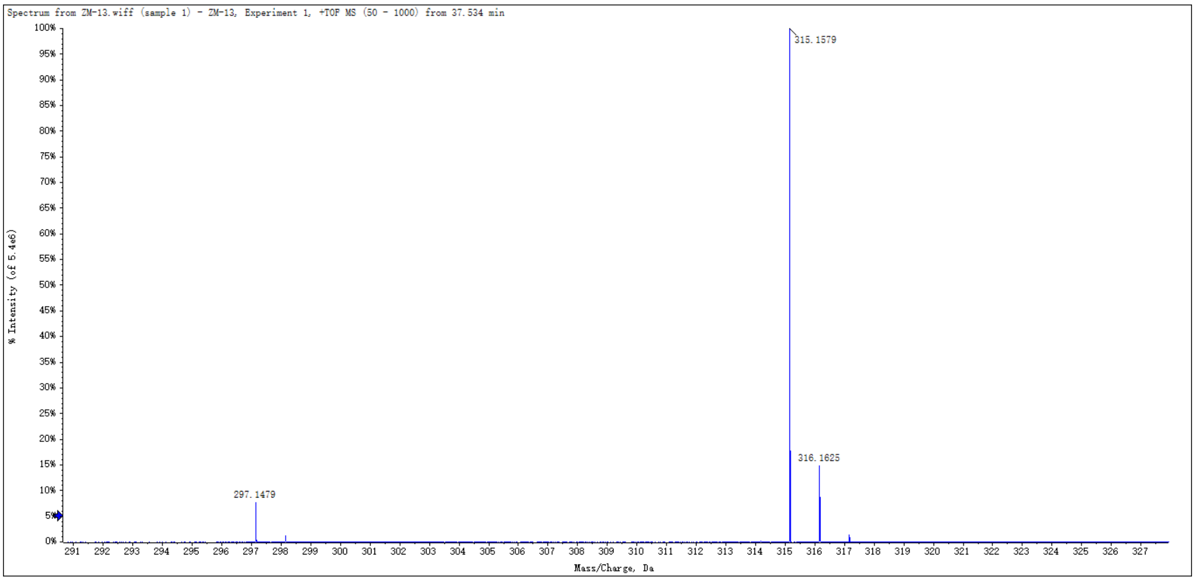

Figure s 18 HRESIMS of tanphenol A (**3**)

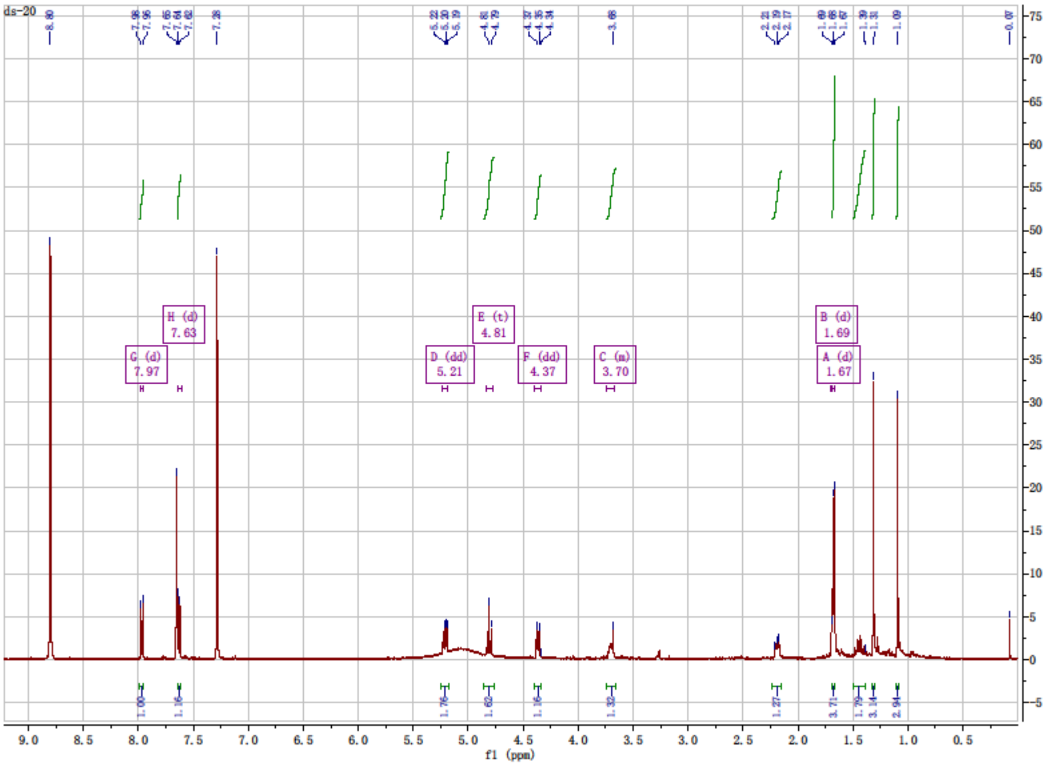

Figure s 19 ^{1}H of tanphenol A (**3**)

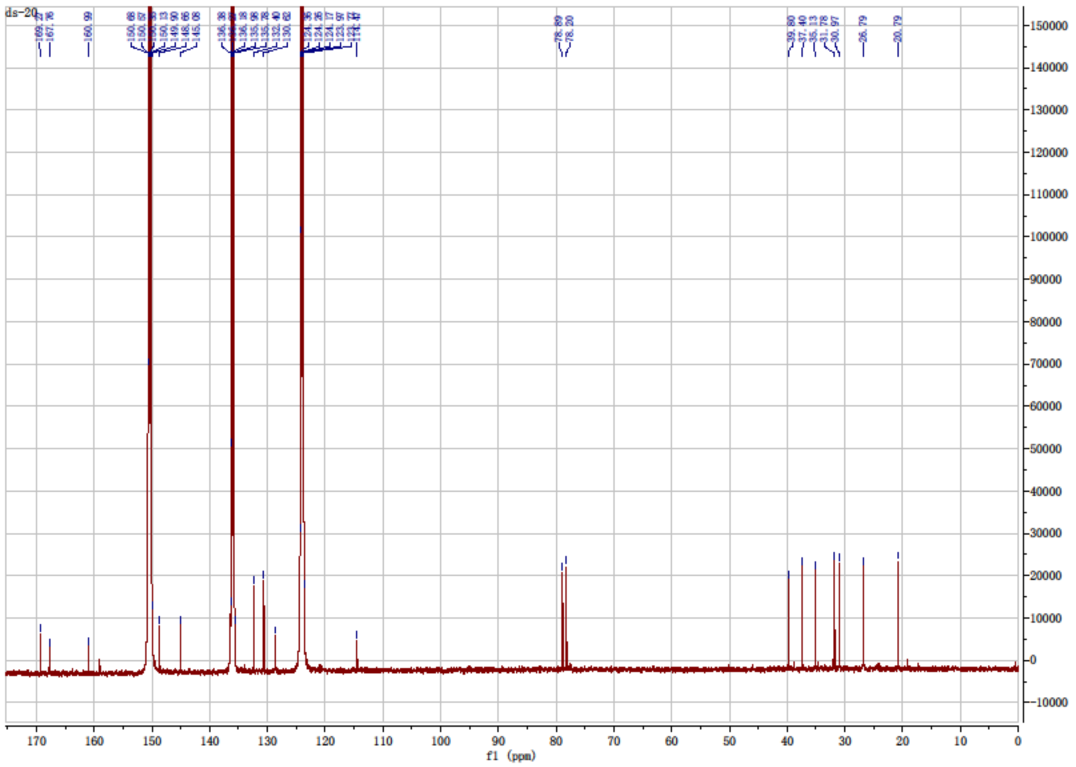

Figure s 20 ^{13}C of tanphenol A (**3**)

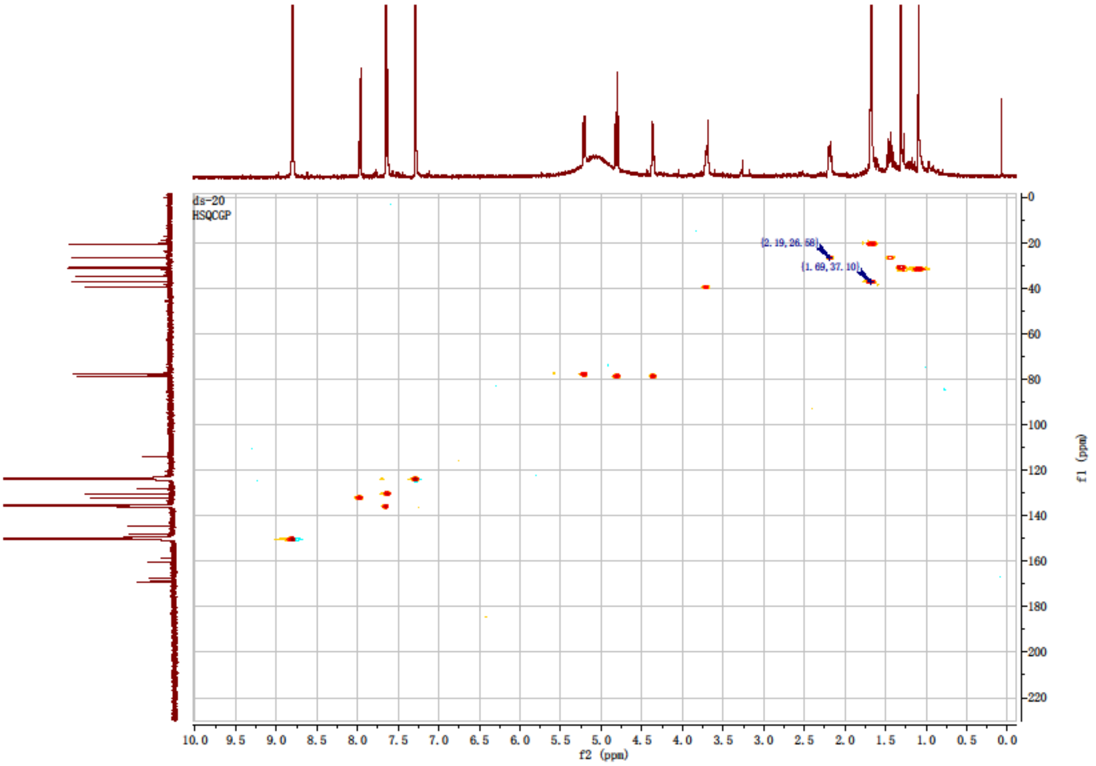

Figure s 21 HSQC of tanphenol A (**3**)

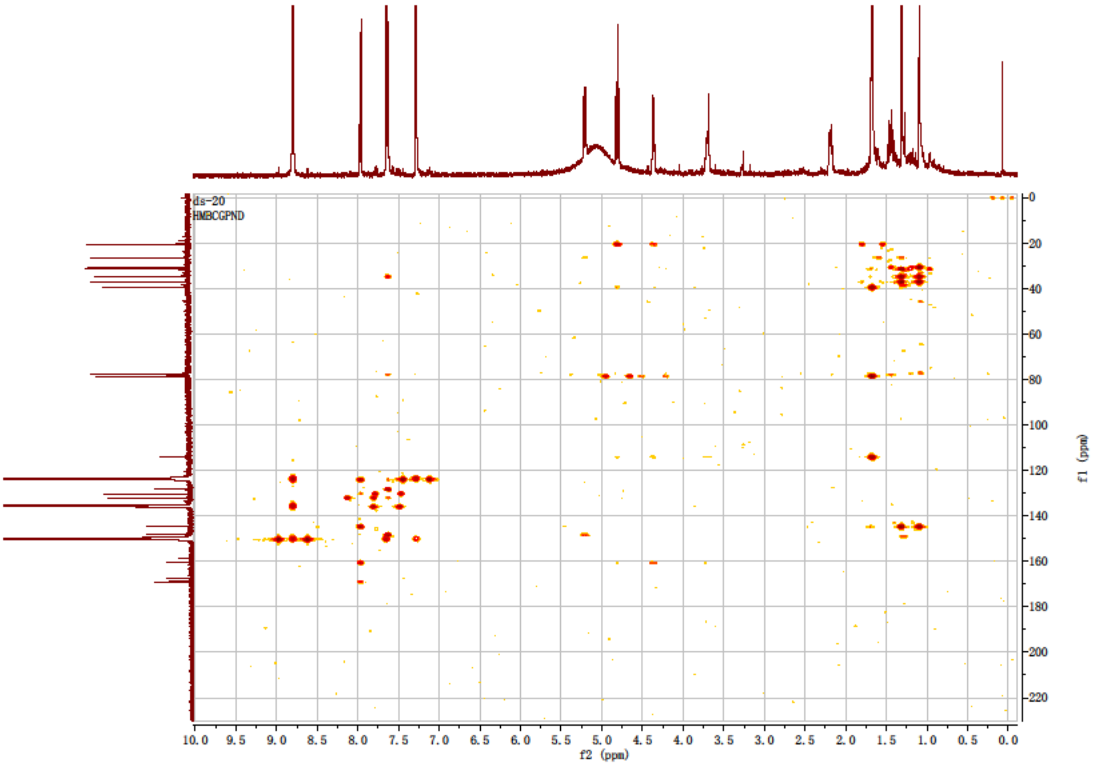

Figure s 22 HMBC of tanphenol A (**3**)

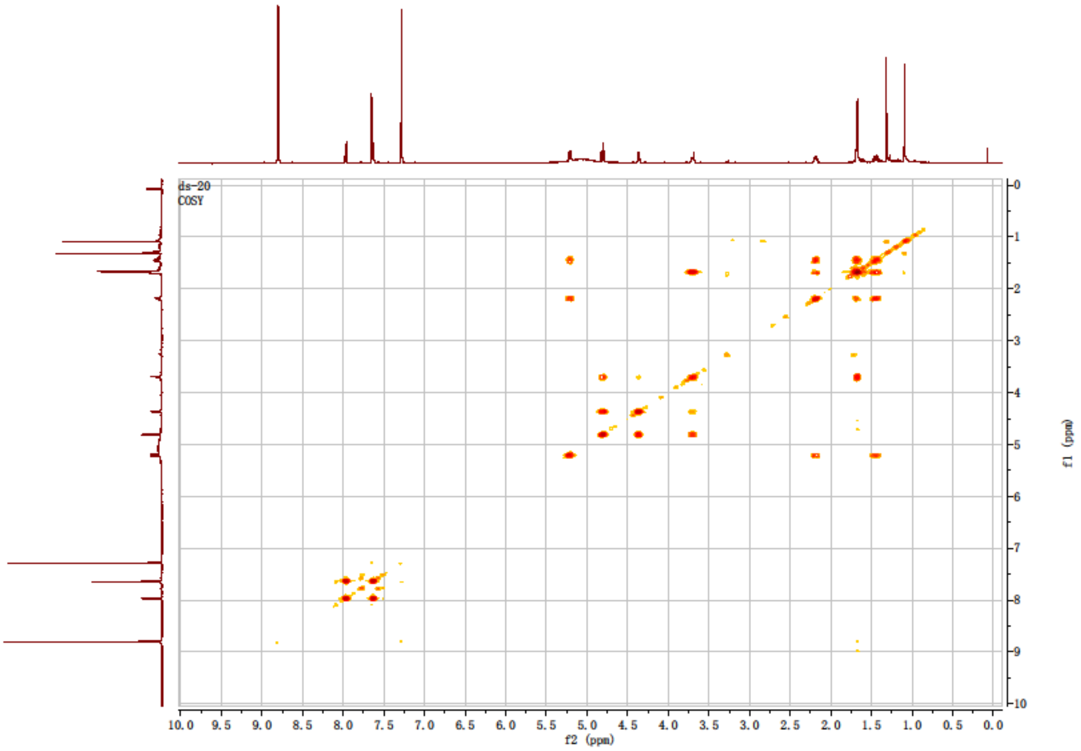

Figure s 23 COSY of tanphenol A (**3**)

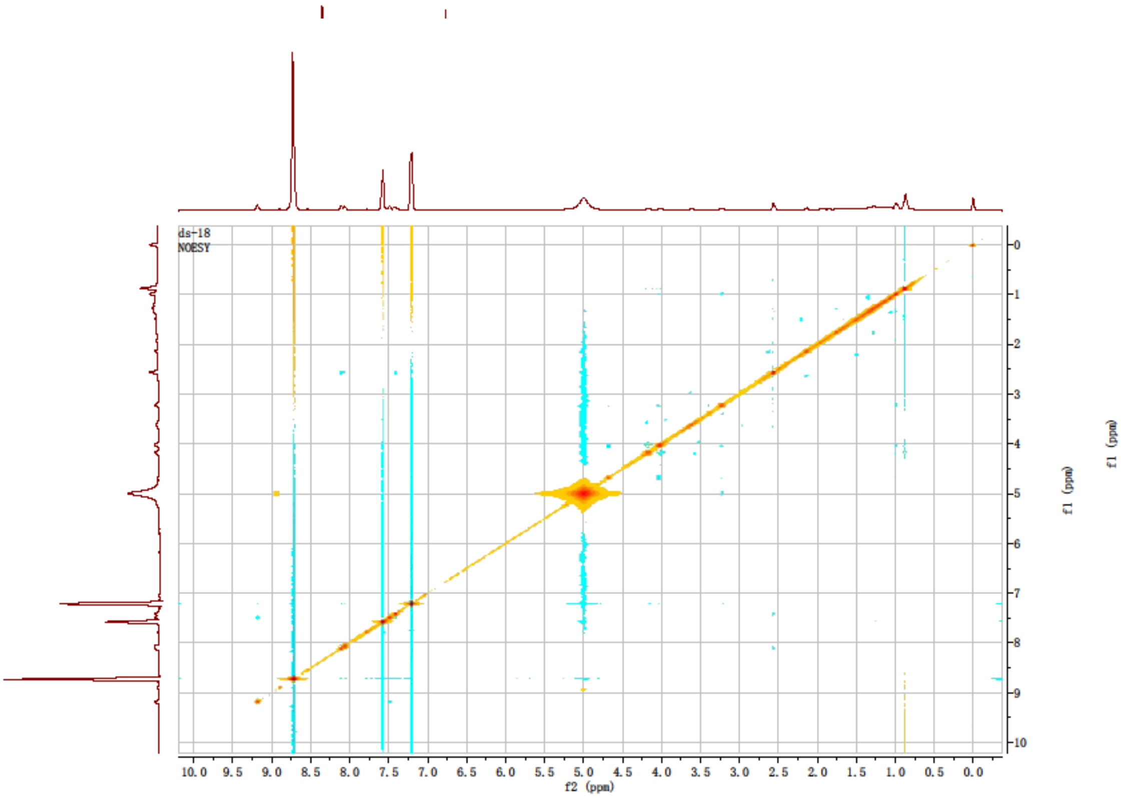

Figure s 24 NOESY of tanphenol A (**3)**

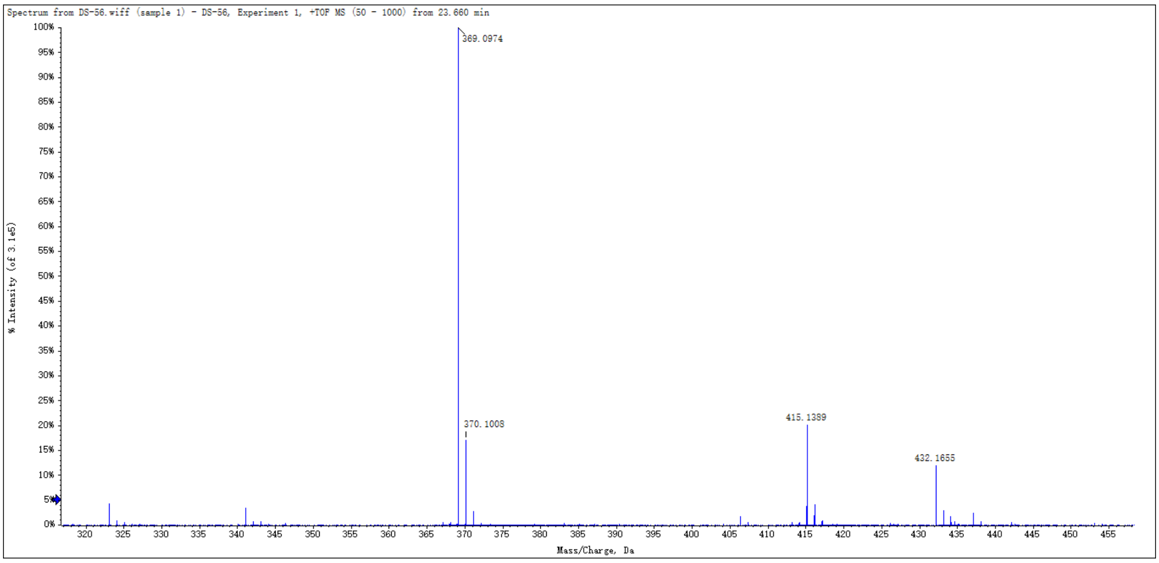

Figure s 25 HRESIMS of diethyl blechnic (**4)**

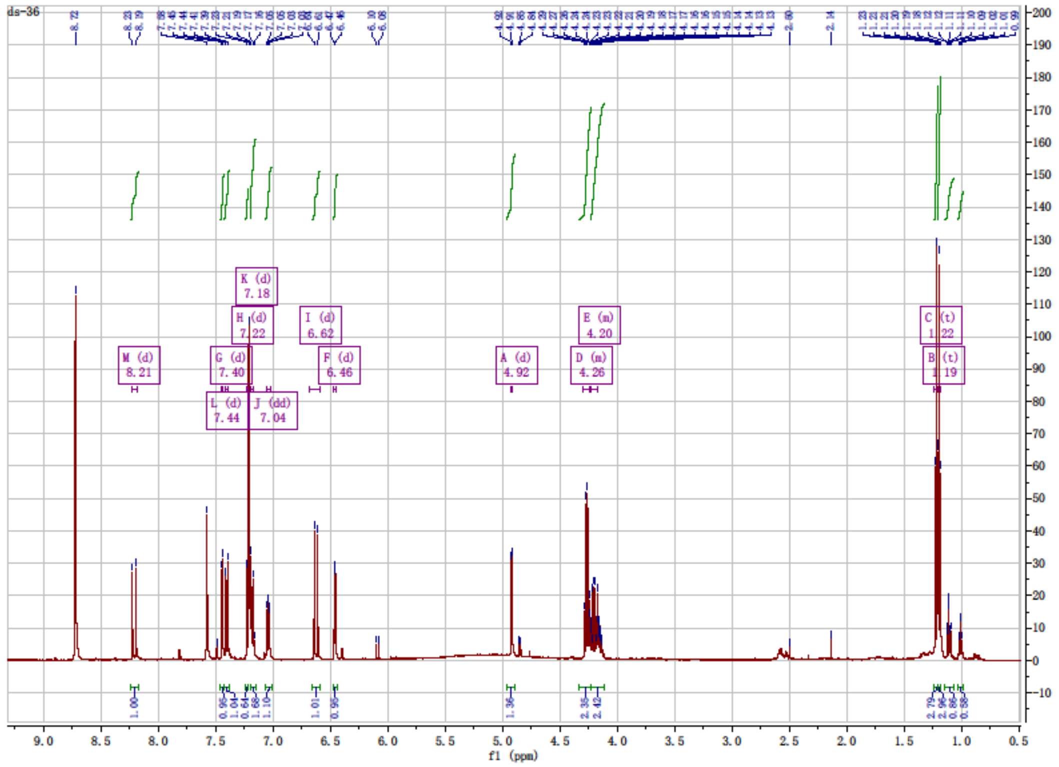

Figure s 26 ^{1}H of diethyl blechnic (**4)**

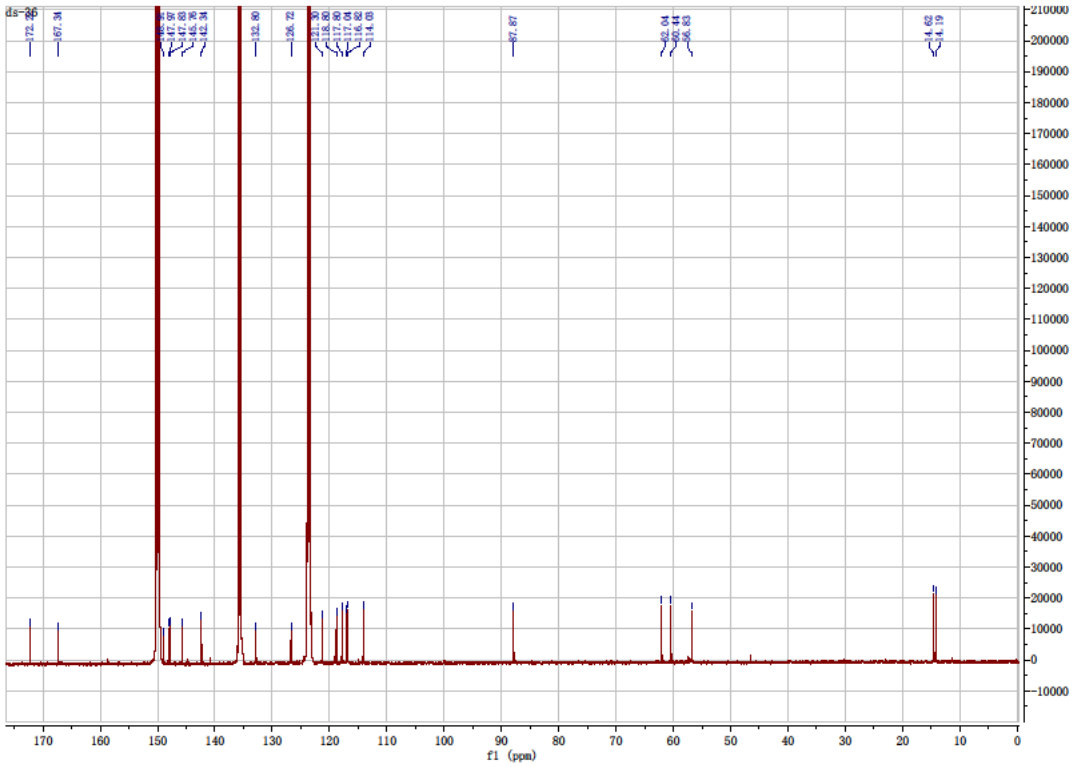

Figure s 27 ^{13}C of diethyl blechnic (**4**)

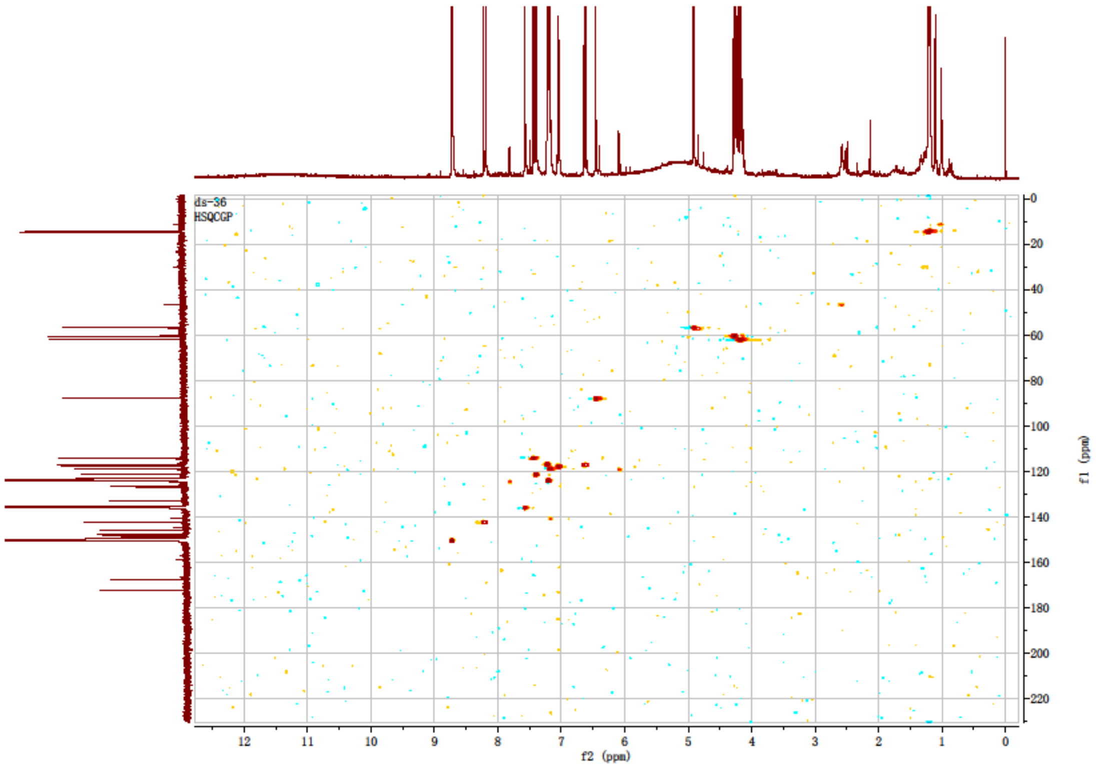

Figure s 28 HSQC of diethyl blechnic (**4**)

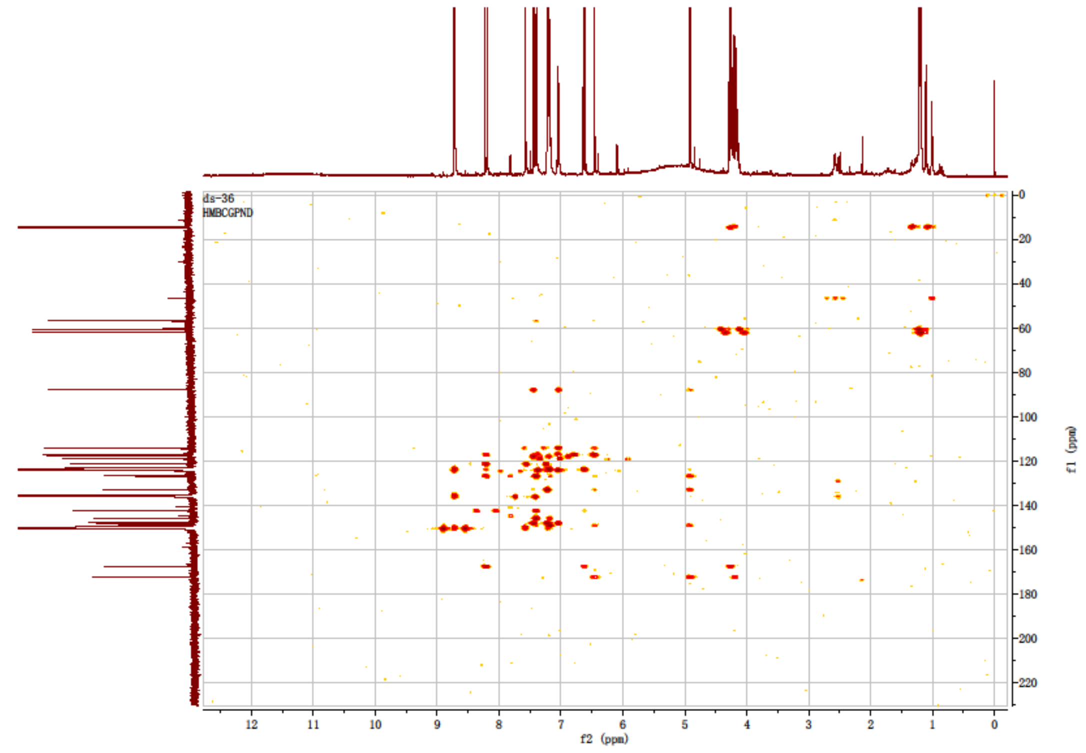

Figure s 29 HMBC of diethyl blechnic (**4**)

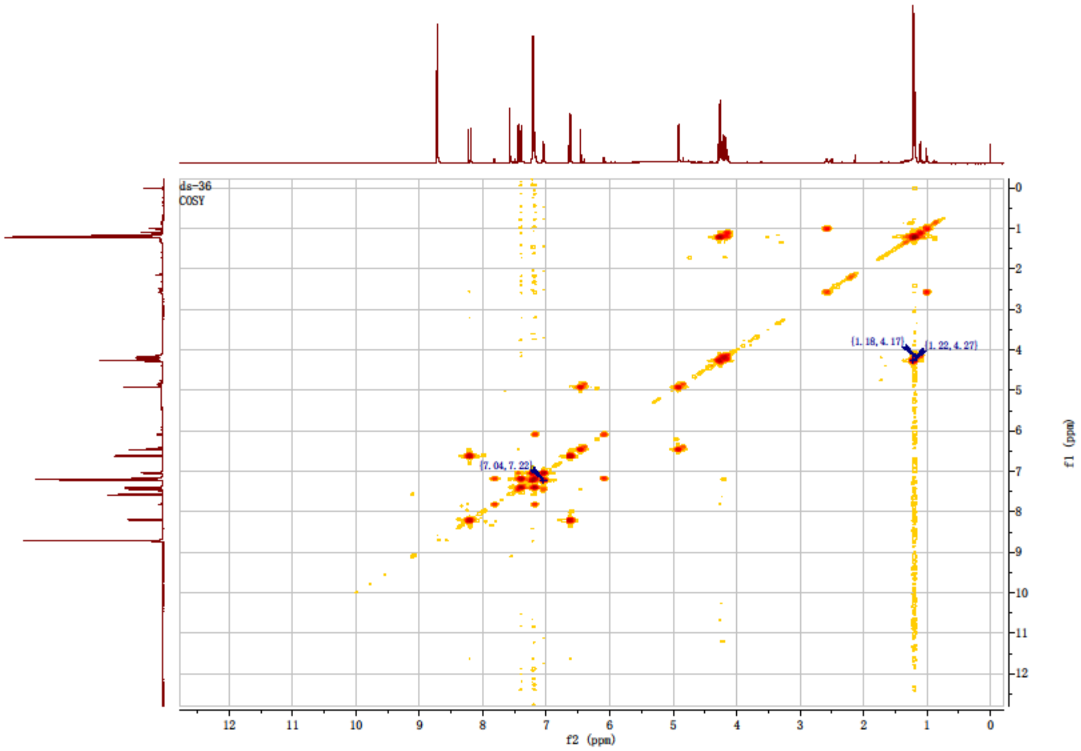

Figure s30 COSY of diethyl blechnic (**4**)

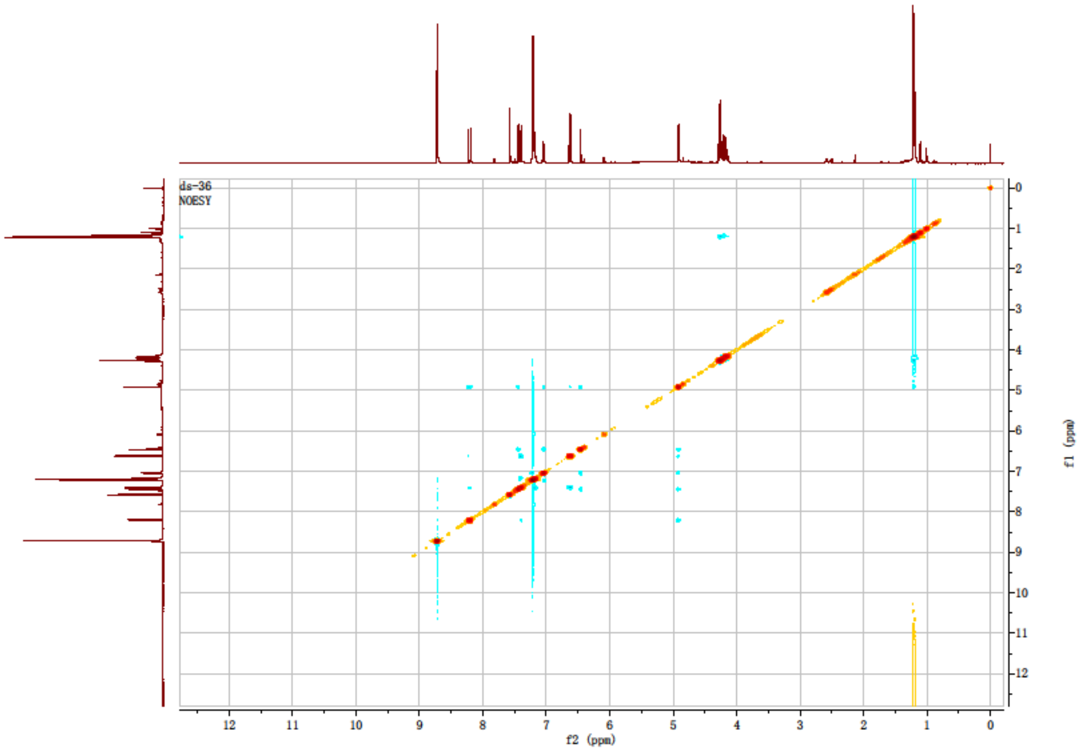

Figure s 31 NOESY of diethyl blechnic (**4**)
